# Identifying value chain trade-offs from fruit and vegetable aggregation services in Bangladesh using a system dynamics approach

**DOI:** 10.1371/journal.pone.0297509

**Published:** 2024-01-24

**Authors:** Dipok K. Choudhury, Gregory S. Cooper, Karl M. Rich, Bhavani Shankar, Sadman Sadek, Nazmun N. Ratna, Suneetha Kadiyala, Mohammad J. Alam

**Affiliations:** 1 Department of Agribusiness and Marketing, Bangladesh Agricultural University, Mymensingh, Bangladesh; 2 International Maize and Wheat Improvement Center (CIMMYT), Gulshan, Dhaka, Bangladesh; 3 Institute for Sustainable Food & Department of Geography, The University of Sheffield, Sheffield, United Kingdom; 4 Ferguson College of Agriculture, Oklahoma State University, Stillwater, OK, United States of America; 5 Digital Green, Dhaka, Bangladesh; 6 Faculty of Agribusiness & Commerce, Lincoln University, Lincoln, New Zealand; 7 London School of Hygiene and Tropical Medicine (LSHTM), London, United Kingdom; Southeast University, BANGLADESH

## Abstract

Significant progress has been made in cereal production in Bangladesh due to an agricultural policy environment that prioritizes the productivity of staple crops over fruit and vegetables (F&V). However, many smallholder farmers remain poorly connected to markets, which may lead to a limited supply response of F&V that can reduce opportunities for sufficient intake in neglected, consumer-facing, smaller retail markets. To address this issue, aggregation schemes have been conceived that collect and transport F&Vs on behalf of multiple farmers. Given the volume of horticultural produce produced and the reliance on developed transport infrastructure, aggregation schemes tend to supply wholesale and urban markets rather than underdeveloped rural and isolated markets. To this end, we investigated how a particular aggregation intervention (‘Loop’) could potentially improve the distribution of F&V to smaller markets whilst improving farmer benefits. We used an innovative system dynamics modeling approach based on Loop`s aggregation services in Jashore, Bangladesh, and to identify the potential trade-offs between consumer outcomes in retail markets and farmer benefits. We find that combining aggregation with a quota at the smaller market, transport subsidy, and current price growth does not result in trade-offs between consumer purchases and farmers`benefits. However, combining aggregation with current price growth can increase demand without losing farmers`benefits. The findings emphasize that standalone and multiple market-oriented interventions generate broader win-win benefits to promote inclusive food systems.

## Introduction

Over the past few decades, significant progress has been made in cereal production (rice, wheat, and maize) to feed a growing population of 165 million in Bangladesh. Currently, 75% of the country’s total cropped area and 58% of total crop production is covered by rice (Alam & Naser, 2020b). However, the UN Sustainable Development Goals (SDGs) have set a target of overcoming all forms of malnutrition by 2030. When viewed through a nutrition lens, the large proportion of dietary energy supplied from this widely produced cereal, especially rice is a major concern due to insufficient micronutrient intake [1]. Bangladesh is ranked 83^rd^ of 113 countries overall and 20^th^ out of 23 Asia Pacific countries regarding food availability, affordability, quality, and safety [2].

Despite progress in alleviating hunger, the prevalence of malnutrition is still high. Nearly 8.9% of the world’s population is undernourished, and malnutrition affects approximately 25.9% of people worldwide [3]. In Bangladesh, it is estimated that 29.5% of the population lives below the poverty line [4], which is associated with severe consequences for productivity, intellectual development, and maternal and infant morbidity and mortality [5].

Around 60% of the total population of Bangladesh suffers from various micronutrient deficiencies, which can lead to serious health problems [6]. In turn, a majority of the population consumes less than 75% of the daily recommended amounts for several food categories [7].

Vegetables represent the largest amount of daily food intake that has an important role in a balanced diet [8, 9]. They provide essential vitamins, minerals, and dietary fiber, as well as feeding the growing population [10]; regular consumption of vegetables further helps meet nutritional requirements in the human diet [11]. However, horticultural value chains, which supply fruits and vegetables (F&V) vital in the combat of chronic diseases and micronutrient deficiencies [12], are frequently characterized by seasonal and perishable supplies, erratic prices, outdated distribution systems, and insufficient awareness around the importance of F&V consumption [13]. The consumption gaps are more pronounced in rural areas, with the rural consumption rate of F&V being about 6% less than the urban rate [10, 14, 15]. Despite higher vegetable production in rural areas, the consumption gap is often driven by: (i) the higher return on produce in the high-demand urban areas due to higher prices; (ii) improvements in transport and storage infrastructure, which have lengthened urban-centric distribution networks, and the comparative economic wealth of urban populations which provide a consumer-base that can spend more on food; (iii) low supply response to rising demands for non-staple foods; (iv) a significant proportion of smallholder farmers not connected to the market, facing insufficient and risky returns [16] which leads to limited diversity of vegetables that can reduce opportunities for sufficient intake [17], and (v) an agricultural policy environment that prioritizes the productivity of staple crops over F&Vs. Therefore, there is a lack of a level playing field allowing farmers to respond to market dynamics [18].

For the past decades, the Bangladesh government has been implementing various initiatives to overcome the above issues [7]. The aims of these initiatives are to ensure farmer benefits and access to the market, whilst providing affordable prices for consumers. For example, market infrastructure has been improved to smooth supply chains, the private sector has been engaged in marketing agricultural products, and the value chain has been strengthened. Modern facilities have been introduced, such as cold storage facilities and improved processing infrastructure to ensure product’s safety, freshness, and quality [19]. In addition, input subsidies and various production-oriented initiatives have been a major focus for the government to increase yield. As a result, vegetable production has been increasing, as well as consumer demand, as farm incomes improve [20].

Most of the time, farmers are overloaded with different types of produce, resulting in issues such as spoilage and loss of quality. Despite recent initiatives, cold storage is rarely available in the wholesale markets of Bangladesh for F&V and is instead used for potatoes, spices, and imported fruits. Small-scale farmers are often neglected in upgrading initiatives such as contract farming, group marketing through subsidized training, and input services due to prerequisites like land ownership and initial investment requirements [21, 22]. documented that integrated cold chain, distribution channel development, and construction of collection and marketing centers in F&V production areas would have improved the farmer`s wellbeing. The Sustainable Agriculture, Food Security, and Linkage’s project implemented by Solidaridad formed producer groups in Khulna and Jashore districts to improve collective negotiation power in terms of input and transport cost reduction regardless of farm sizes [23].

Aggregation schemes, which collect and transport agricultural produce on behalf of multiple farmers [24], provide one such market intervention that can mitigate the lack of market integration, and high transportation costs to increase consumption and the nutritional intake of vulnerable groups [13, 23]. However, given the volume of horticultural produce created by pooling multiple farmer loads and the reliance on developed transport infrastructure, aggregation schemes may preferentially supply wholesale and urban markets rather than underdeveloped rural and isolated markets [25, 26]. Given these constraints, our study aims to identify to what extent aggregation and marketing schemes can benefit farmers whilst also improving the availability and affordability of vegetables in smaller, rural, and neglected markets. We based our study on Digital Green’s “Loop” aggregation program in Jashore [27], Bangladesh.

Jashore has topped the areas producing the most vegetables per hectare a year. Farmers of Jashore produce the highest 25 tons per hectare per year compared to the country’s average of 22 tons per hectare of F&V [28]. In addition, most 33 types of commercial vegetables are grown in Jashore District and supplied to urban areas markets. The farmer produces a lot in Jashore, but the connectivity with the urban market has always been an issue. Until recently, using a ferry to cross River Padma was the only way to travel to the Urban market. Ferry service often gets disrupted during winter vegetable production due to fog. This combination of factors motivated Digital Green to choose Jashore as a location for the Loop program. In addition, as the Loop aggregation scheme is an FtF (Feed the Future) project, it should be implemented in the FtF zone of Bangladesh. Jashore is one of the 20 FtF districts in Bangladesh where particularly USAID-funded activities have been implemented. Given the above context, we considered the Jashore district as a suitable case study to understand how the aggregation scheme may benefit both the market access of horticulture producers and the availability and affordability of F&V in a consumer-facing market in the district.

Against this backdrop, we applied a system dynamic modeling approach to identify the types of interventions and policies able to increase the delivery of F&Vs towards smaller markets in the Jashore district whilst preserving the farmer-facing benefits of aggregation participation. Based on a dataset with daily transactions of F&Vs sold by the aggregators and a participatory group model-building exercise, we develop a set of future potential scenarios. The future scenarios explore prospective changes to aggregation and the improvements to the marketing environment required to encourage smallholder farmers to become more sensitive to fresh produce availability and affordability for nutritionally insecure consumers. We then combine the multiple interventions to increase the local availability and affordability whilst also attempting to secure farmer income and the production of fresh produce.

## Study context

**Fig 1:**
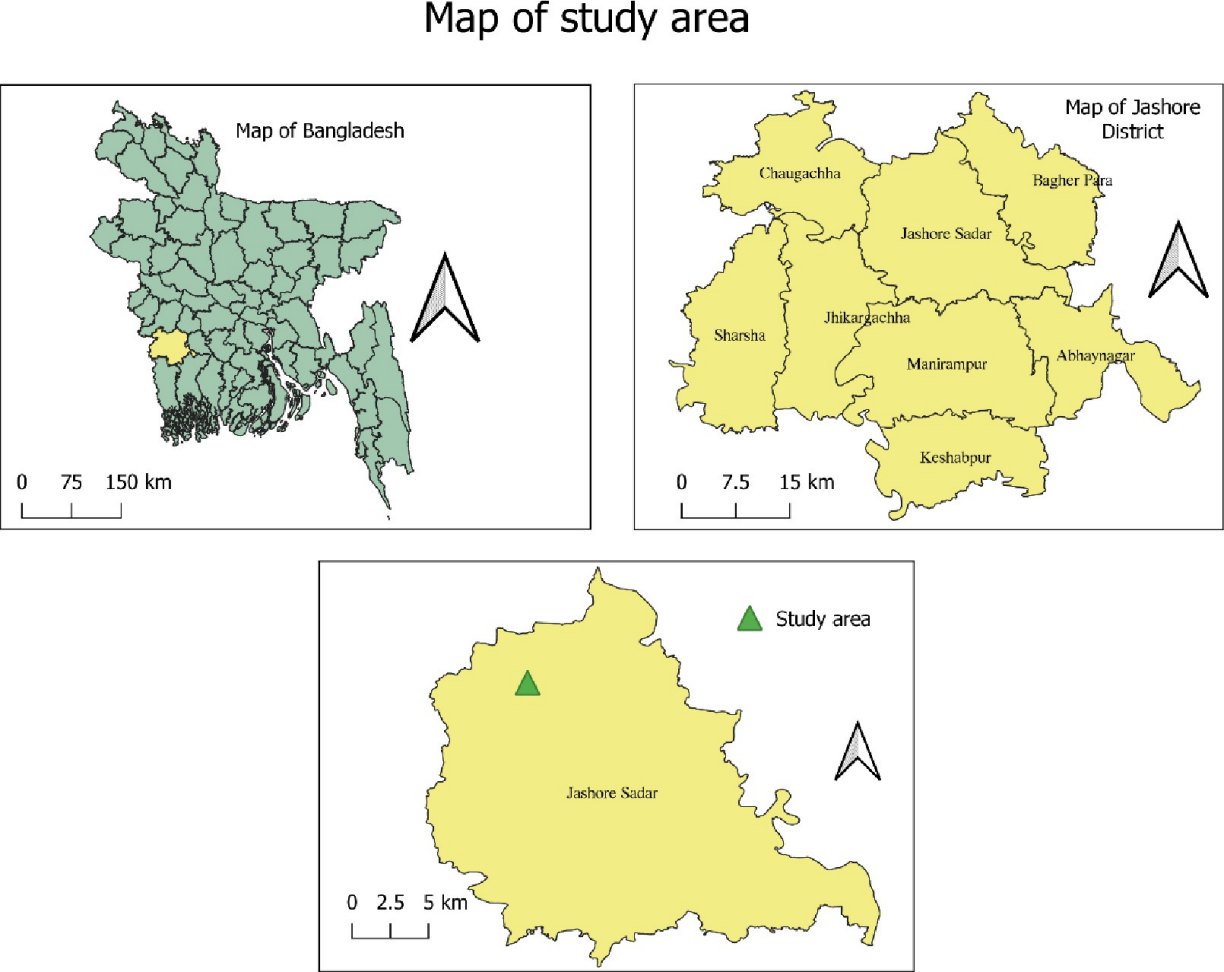
Map of Bangladesh (left), Jashore district (left), and Jashore Sadar (below), where this study was conducted. Source: https://data.humdata.org/dataset/administrative-boundaries-of-bangladesh-as-of-2015.

Jashore (Fig 1) is situated in the southwestern region of Bangladesh and is the 13^th^ largest among the total of 64 districts in terms of administrative area. The estimated population of Jashore is around 2.8 million, with 20.3% of the total population employed in agriculture. Jashore is unique among the districts in Bangladesh because of its ecological and geographical situation. Owing to tremendous economic growth and evolving vegetable cultivation practices, Jashore is one of the zones that has converted from subsistence to commercialization farming practices [4, 29, 30]. Presently, Jashore contributes approximately 5% of national vegetable production, ranking eighth among the 64 districts [31].

Despite increased production, rural vegetable consumption is still lower than FAO recommendations, owing to a fragmented supply chain with 84.4% of farmers classified as either small or marginal [31, 32]. In addition, markets in Jashore are unregulated and have a long forward chain, with value-added distributed amongst a multitude of actors. Due to fragmented farming, individual farmers hire their own transport and take small quantities of products to market, thus lacking the collective bargaining power to negotiate better prices. Moreover, on average, it is estimated that 15% of vegetables are damaged *en route* to the market due to poor transport and storage facilities [33, 34].

In response to these limitations, Digital Green, a non-government organization working in Bangladesh and India introduced the “Loop” aggregation scheme in the Jashore district, covering 30 villages, in April 2017 to optimize transport logistics and increase smallholder profitability [27]. In 2017–2018, about 3,020 hectares of land were cultivated solely for vegetables, producing 77,199 tonnes in Jashore Sadar. The proportion of Loop F&V is 8% in the 2017–2018 Rabi season and 13% in 2018–2019 of the total production of Jashore Sadar [31]. As of 2018, there were approximately 27,000 farmers across the four unions of Jashar Sadar, of which 4302 were loop farmers (As captured by the model). Digital Green selected aggregators with smartphones to record the sales transactions for each village. These aggregators collect vegetables daily fresh from the farm, identify the potential best market in which to sell, manage market transport and sales, and distribute payments back to farmers through a mobile application with a digital receipt. The farmers participating in aggregation receive their market revenue after deducting the transport cost (normally BDT 0.5-1/Kg) and service charge (BDT 0.25–1.00/Kg) (1 USD = 84 BDT). Loop farmers are free to decide if they would like to supply to only aggregators or also to non-Loop market actors, or they can transport vegetables to local markets themselves on any given day.

In Bangladesh, the same markets cater to all kinds of F&V. Hence, while aggregating and going to a market, LOOP vehicles usually contain a different kind of F&V. However, there is little variety of F&V in each season within the village. So, it is primarily three to four major types of F&V in one vehicle, plus some F&V from the homestead garden given by the rural women. Each farmer’s produce is packed in his or her sack, so there is no mixture of products by different farmers. While Loop promotes sorting and grading to its client farmers, there is no formal requirement or process of sorting produce by freshness, color, brightness, and so on. During the aggregation process, each farmer’s sack is weighed and sold as it is to the target markets.

## Data and methods

### Data sources

A set of six data sets were used in this analysis; these are summarized in Table 1 and described in detail below.

**Table 1:** Description of six datasets and their function in the model.

Dataset	Data type	Period	Sample size	Role in model design and evaluation
Rapid value chain analysis	Qualitative/Primary	November, 2018	28 interviews related to the vegetables value chain	Function of actors in vegetables value chain system in Jashore
Loop dashboard-Jessore Sadar	Quantitative/ Secondary	April 2017-January, 2019	82,312 individual market transactions	Daily time-series of Loop-farmer joining, drop-out, price, quantity, market, transport cost.
Farmer household survey	Quantitative/Primary	September, 2019	360 farming households (120 Loop and 240 Non-Loop)	Loop and non-Loop- market participation, consumption, wastes, given away, price, commission.
Value chain’s actors survey	Quantitative/Primary	September, 2019	40 value chain actors	Actors’ trading, capacity, commission, wastage, price, transport.
Spatial group model building sessions	Qualitative and quantitative/Primary	May-June, 2019	Eight sessions in four days (nine farmers, eight aggregators, four wholesalers)	- Drivers of adoption, market choice and traders’ choice • Transport costs • Vegetables rout in • Market power • Governance • - Scenario testing
Reference group meetings	Qualitative and quantitative/Primary	November 2019	Two sessions in one day (four DAE^a^, three BARI^b^, one BADC^c^, one aggregator cum farmer, one trader, one academic researcher)	Some initial model findings were shared with stakeholders and their feedback was used to revise the model

^a^ Department of Agricultural Extension.

^b^ Bangladesh Agricultural Research Institute.

^c^ Bangladesh Agricultural Development Cooperation.

We first collected data on the structure of the system from a rapid value chain analysis of the vegetable value chain in the Jashore district. The RVCA included all actors in the chain, encompassing farmers, aggregators, wholesalers, retailers, commission agents (“Aratdars”), and consumers. Our 28 semi-structured qualitative interviews covered household information, production decisions and market choices, trade dynamics over space and time, upstream and downstream flows, wastages and quality, nutritional awareness, and consumption habits.

Second, the Loop dashboard dataset maintained by Digital Green (preview available here: http://www.loopapp.org/loop/analytics/) is a record of farmer vegetable supply dates, types of vegetables supplied, selling price, market revenue, transportation type used and cost, the participation of female-headed farming households, and village and market information. Covering April 2017 to January 2019, these 82,312 individual market transactions were processed and used in the SD model, including daily time-series data for farmer participation, market supplies, and prices received. This covered a total of 17,400 tons of fruit and vegetables sold by 30 aggregators at 13 markets, including local and distant markets outside of the Jashore district. In total 4,302 farmers engaged in this Loop aggregation scheme from 30 villages across Jashore. Among the traded vegetables, 95% traded at nine local markets (3–10 km radius) and 5% at four distant markets (outside of the Jashore district market). It was also noted that the local market was dominated by the major *Satmail* market, where 81% of vegetables were traded and the daily supply in the Rabi season was around 40,000 kg.

Third, to further increase the volume of quantitative data available to parameterize the model, we collected data based on 360 farmer household surveys, including 120 Loop households and 240 non-Loop households in the Jashore district. We first selected two pre-specified Bazars, to ensure that we collected data from a range of market and village environments: Satmail Bazar and Lebutala Bazar, the largest and the smallest market respectively based on the total volume of market transactions in the Loop dashboard. We chose two Loop villages, and two paired non-Loop villages within a pre-specified radius of the Loop villages from each of the two selected markets. Regarding the sampling of households, we randomly selected 30 Loop farmers from the database of Loop farmers who had carried out vegetable transactions within the last 12 months from each Loop village. We further selected 30 non-Loop farm households growing vegetables for sale from the same village. To increase the robustness of comparisons between Loop and non-Loop farm households, we also selected 30 farm households from each of the non-Loop villages. Non-Loop households were selected randomly from the list of farm households provided by the Department of Agricultural Extension.

Fourth, we conducted a second round of quantitative surveys of value chain actors along the vegetable value chain, including 32 actors in local, distant, rural, and urban vegetable markets. We conducted quantitative surveys of value chain actors along the F&V value chain, including 32 actors in local, distant, rural, and urban vegetable markets. Of them, 4 aggregators, 8 Aratdar, 8 distant wholesalers, 4 local wholesalers, and 8 were retailers. We selected randomly these different actors based on Loop F&V flows to actors across the value chain.

We focused on collecting data on vegetable trader capacities, destinations, modes of transport, and cost, as well as associated commissions, wastages *en route*, quality, and consumer consumption patterns.

Fifth, we conducted a series of spatial group model building (SGMB) exercises, with a total of eight sessions on four different days in Jashore with stakeholders along the vegetable horticultural value chain. SGMB uses participatory GIS techniques to identify the temporal and spatial dimensions of the value chain alongside system thinking principles [35, 36]. In particular, SGMB utilizes the offline GIS platform “LayerStack” to facilitate discussion between value chain stakeholders with regard to the location of system stocks (e.g., market and storage), feedbacks (price and demand), and delays (e.g., seasonal investment). Session agendas, timings, participation, and materials were planned and prepared in advance [36]. Each session was planned to last three hours, involving a diverse group of stakeholders including male and female farmers, aggregators, and wholesalers within the Jashore vegetable value chain. A range of qualitative and quantitative data were derived from the SGMB sessions, including an extensive model (summarized in the next section and SI) that was used in the scenario analysis.

Finally, we shared our initial modelling results in a series of reference group meetings to seek feedback from practitioners, academics, researchers, and policy makers in the horticultural system in Jashore through discussions with the reference group.

### Model description

The system dynamics (SD) method was introduced to enhance in learning complex system, is fundamentally and interdisciplinary science, is grounded in the theory of nonlinear dynamics and feedback control and draws on cognitive and social psychology, economics, and other social sciences to incorporate human dimensions and decision making [37].

In SD modelling approaches is used to applying five general steps which are (1) problem articulation involves determine boundaries, variables, time horizons, and data sources, (2) development of a dynamic hypothesis pertaining to the initial explanation of the endogenous dynamics of the problem at hand, (3) Formulation of a simulation model which move from qualitative to quantitative understanding of the problem (4) Testing the simulation model aims to build confidence in the quantitative model, and finally (5) Policy/strategy design and analysis which identifying leverage or tipping points of the system [38].

In system dynamics modeling, the key variables and concepts are expressed through stocks, flows, feedbacks, and delays which are all linked together via a series of differential equations [37]. In system dynamics, feedback loops, delay and nonlinearities are captured explicitly [39]. Furthermore, it investigates the complex food system challenges including the design of inclusive value chain intervention. For our system dynamics model [36, 40], the structure, parameter values, and equations of the model were informed by six datasets (Table 1), which are all evaluated for their reliability in S1 and S2 Files.

We developed the system dynamics model using the modeling software STELLA Architect (ISEE Systems, version 1.8). In order to capture both short-term decision-making processes (such as daily aggregation participation and price) and long-term outcomes (such as production and consumption), each simulation was run with daily timesteps. The model was parameterized over the first 308 days (i.e., April 27, 2017, to February 28, 2018), with the next 232 days (i.e., March 1, 2018, to October 19, 2018) reserved for model evaluation. Alternative scenarios were then activated from October 20, 2018, and run for the next five years (until December 31, 2023) to assess the effectiveness of alternative interventions on different outcomes of interest (Please see S5 File for the full equations underlying the model). In line with the aims of this study, we modeled total vegetable availability and affordability by aggregating the total daily volumes of all 42 types of vegetables found in the Loop dashboard. Below, we briefly discuss the modelled structure, feedbacks, decision-making processes, and drivers of vegetables supplies across the value chain in Jashore Sadar, Bangladesh.

**Fig 2:**
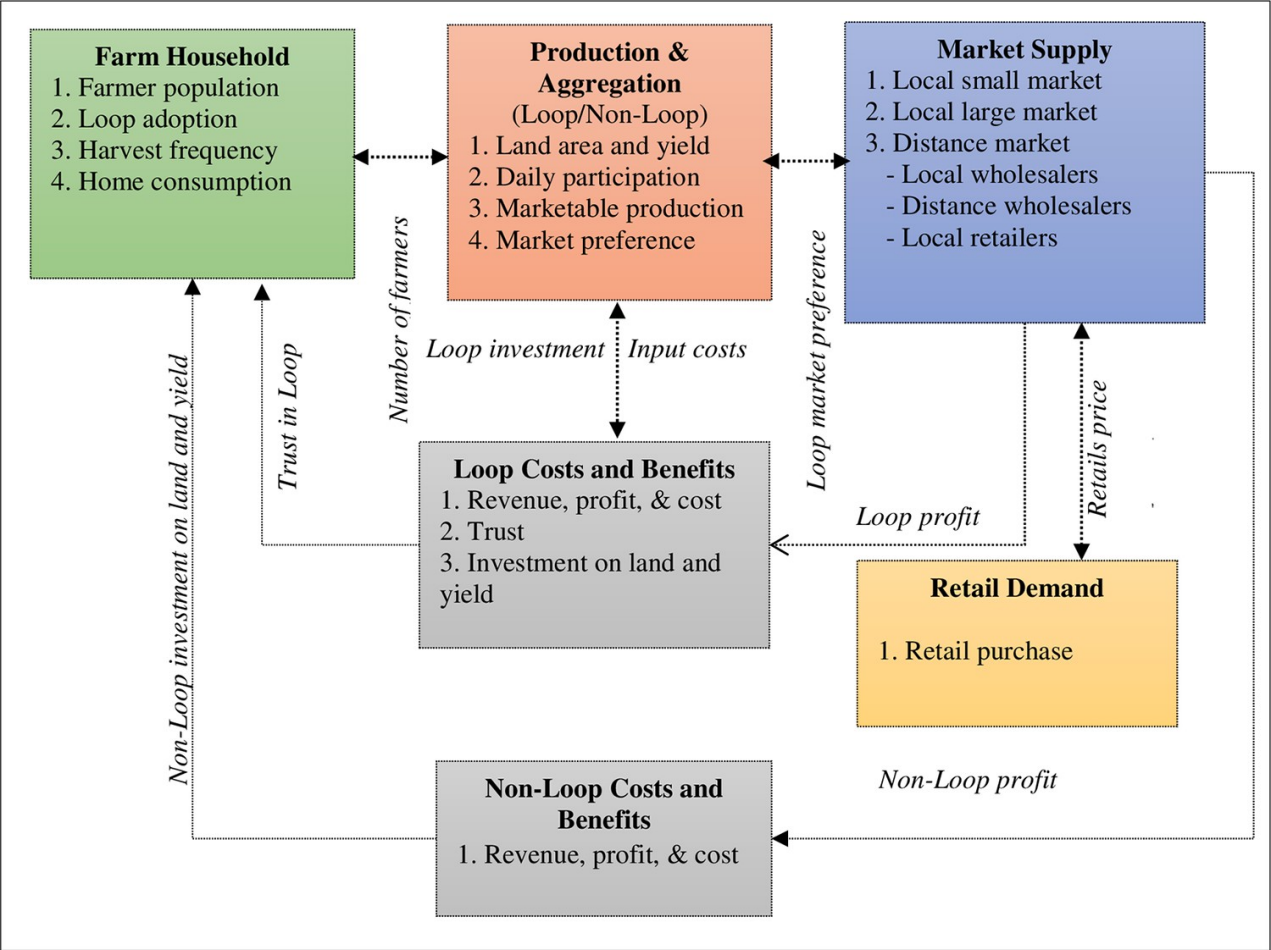
Structure of the model and the interlinkages between different modules.

The model comprises six major interactive modules: farm household; production and aggregation; market supply; Loop costs and benefits; non-Loop costs and benefits; and retail demand (Fig 2 and Table 2).

**Table 2:** Description of the casual dynamics and decision-making drivers captured within the model.

Module	Decision	Casual dynamics	Data sources
Farmer household	Loop adoption	i) Loop extension efforts ii) Word of mouth among farmers iii) Loop trust	i) Survey data ii) [61] iii) [62]
	Farm household consumption	Baseline rate from household survey (4% of annual production).	i) Household survey
Production and aggregation	Harvest frequency	i) Both Loop and non-Loop populations harvest and sell once every 3 days in Rabi (October -March), once every 5 days in Kharif-2 (June -October), and once every 5 days in Kharif-1 (March—June).	i) Household survey ii) SGMB data
	Vegetables given away (in kind, gift)	i) Baseline rate from household data for both populations (0.06% of total yield)	i) Household survey
	Post-harvest wastage	i) Baseline rate from household data for both populations	i) Household survey ii) SGMB data
	Daily Loop participation	i) Short-term satisfaction depends on trust, driven by profit and sales guarantees	i) SGMB data
Market supply	Market choice	i) Market profit from sales ii) Guaranteed sales iii) Market trust iv) Market costs	i) SGMB data
	Number and types of markets	i) Market split into 4 locals small, 2 local large, and 2 local distance markets	i) Dashboard data ii) SGMB data
	Season- based number of traders	i) Number of traders in Rabi, Kharif-2, and Kharif-1 ii) Traders (retailers)in local consumer markets: Rabi (110), Kharif-1 (90), and Kharif-2 (100) iii) Traders in small local markets: Rabi (35); Kharif-1 (20) and Kharif-2 (25). iv) Traders in large local markets: Rabi (160), Kharif-1 (120) and Kharif-2 (130).	i) SGMB data
Retail demand	Retail price at the local consumer market	i) Per customer daily purchases increase if the local consumer market (LCM) price is less than the reference price (Tk. 20/kg) and decreases if the reference price increases ii) The price elasticity of demand for vegetables is linked to the rate of demand changes with the rate of price changes.	i) [37] ii) [63]
Benefits and costs	Investment in land and yield	i) Rate of investment in land improvement and yield which is 2% of cumulative profit	i) SGMB data ii) Survey data iii) Modeler intuition

#### Farming population

The total number of farmers in the model is 25,000—split between Loop and non-Loop farmers, with a ratio of 1:6 based on dashboard data and the Department of Agriculture Extension (DAE), Bangladesh. The figure for the overall farmer population is based on the government`s 2011 population census. The transition of non-Loop farmers to Loop farmers is based on the [41] diffusion model, and adoption is driven by community extension efforts and the benefits of the technology, which are spread through word of mouth (Table 2). We extended this model by connecting membership of the aggregation scheme to the expected Loop profits from sales on any given day. Loop farmers start to supply through Loop after the adoption of the intervention, whilst non-Loop farmers must supply to the market themselves on any given day.

#### Production and aggregation

The production and aggregation module were constructed based on the seasonal (i.e., Rabi, Kharif-1, and Kharif-2) yields, land area under vegetable cultivation, and number of Loop and non-Loop farmers. Seasonal supply frequency was the same irrespective of Loop participation in the model and was derived from the Loop dashboard and household survey data. Aggregation happens in Rabi three times, Kharif-2 five times, and Kharif-1 five times a week, both for Loop and non-Loop farmers. Seasonal-based yields were generated from per decimal land under vegetable cultivation based on the DAE, SGMB, and household survey data. Yields shifted from a maximum of 105 kg/decimal in Rabi to 80 kg/decimal in Kharif seasons. Land area under vegetable cultivation depends on seasonal farming. Based on SGMB and DAE data, farmers`maximum land holding occurred in the Rabi season, followed by Kharif-2 and Khairf-1, when farmers cultivated their commercial vegetables.

The production and aggregation module were then divided into i) household consumption of F&V production; ii) F&Vs given away (in-kind payment, gift); iii) farm wastage; and iv) the remaining quantity of Loop and non-Loop vegetables flowing downstream towards the market. In addition, we found from SGMB data that Loop farmers would self-supply the market when the aggregation was above capacity, i.e., above 2000 kg/day, which was set in the model based on the Loop dashboard data; therefore, in such circumstances, the model adds the excess aggregation to the non-Loop market supplies.

#### Market supply

Markets in the developed model were divided into three categories [local large market (LLM), local small market (LSM), and local consumer market (LCM) in Jashore Sadar] based on SGMB and Loop-dashboard data. The volume of vegetables supplied to the market depends on seasonal variation. Usually, the supply capacity of farmers during Rabi is higher than in the Kharif-1 and Kharif-2 seasons. According to Loop dashboard data, 80% of vegetables are sold through LLM market, with traders then more likely to supply produce to distant markets like Dhaka and other nearby districts of Jashore. The number of farmers and supply capacity and traders are less in LSMs than in LLMs. In LCMs, local retailers purchase vegetables and sell them directly to consumers. According to SGMB and RVCA findings, the capacity per trader in the LLM market was parametrized as 4,000 kg/day with an average of 130 traders and, 1,400 kg/day with an average of 30 traders at each LSM. The model, however, showed retailer capacity for selling on each day to be an average of 362 kg/day, and consumer purchases set at 3 kg/day based on household and SGMB findings.

The proportion of Loop market supplies sent to each market depends on the market preference of each farmer population, which is driven by the price, profit, and transportation costs. According to SGMB data, we parametrized the default proportion as 80% in the model in the study area for LLMs, with the remaining proportion distributed at 10:10 among LSMs and LCMs based on market preference shown by the model itself for Loop. The proportions fluctuated based on the model output of market preference. Non-Loop ratios were set as constant in the model, i.e., 80%, 10%, and 10% for LLMs, LSMs, and LCMs, respectively.

#### Farmer benefits

First, market-wise total farmer revenue was calculated as the volume of total vegetables outflow multiplied by the trader’s buying price on a given day. Second, we calculated per farmer revenue as the total revenue divided by the number of farmers supplying each market. Third, per farmer profit at each market was calculated by subtracting all daily costs (i.e., inputs for production, transport, market fees) from per farmer revenues. Fourth, we calculated weighted per farmer profits per unit (Kg) by aggregating per farmer profit per unit (Kg) at each market on a given day based on the proportionate of the different markets. Weighted per farmer profit per unit (Kg) then drives trust in the Loop aggregation scheme as farmers and traders build successful transactions within a certain period of time (30 days long transaction). Trust in each market (LLM, LSM, and LCM) is parameterized based on inventory and farmer seasonal supply frequency. Inventory is determined by the market stock and market outflow of F&V. This trust will influence the supply of F&V direction, even if LSM or LLM generate higher prices but the farmer will supply LCM in the short run[42]. Trust then feeds-back to influence Loop adoption, daily aggregation participation, and aggregation quantities.

In the model, each farmer’s production costs (e.g., seeds, fertilizer, irrigation, pesticides, insecticides, manure, hormones, tractors, and rotavator) were randomly sampled from a distribution parametrized by the household survey data (i.e., normal or log normal distribution), which were then summed to calculate daily costs per farmer. Daily profit accumulated as a stock over time, which farmers can use to invest in their land and production at the start of each season. Based upon our discussions with participants during the SGMB and RVCA, if these accumulated profits are adequate and growing, then farmers should be able to invest 10% of their profit in land expansion, and it is possible for them to invest 1% for better-quality input to increase the yield by 10% over time.

Consumers who do not produce F&V instead purchase them for their home consumption from the retail market (i.e., LCMs) as opposed to LLMs and LSM wholesale markets. Consumer purchases on any given day are driven by the selling price of F&V in the LCM. The rate of demand change is smoothed under the assumption that individual consumers purchase once every three days. To assess their reliabilities and implications for model behavior, the elasticities and smoothing coefficients underwent qualitative evaluation.

As per SGMB data, we set the retailer numbers in the model based on seasonality as the numbers fluctuate over time. Per retailer, demand was generated by the number of customers per retailer multiplied by the per-customer demand and feedback with the volume of F&V entering the LCM.

### Model evaluation

The purpose of building a system dynamics model is not to forecast future conditions but rather to explore the system dynamics underlying a problem [37, 43–45]. To this end, we conducted two evaluation process (S1 and S2 Files) to assess the model`s reliability and its credibility, namely: (i) behavior reproduction, and (ii) qualitative parameter assessment.

#### Behavioral reproduction

Behavioral reproduction is one of the tools used to understand the disparity between the model and observed data. Descriptive statistics are the most common method to assess the disparity [37]. Whilst the LLM supply timeseries was a major driver of the model`s construction, we applied this evaluation process to assess the population, aggregation and daily farmer participation trends, which were reserved for the purpose of independently evaluating the model’s abilities to replicate historical dynamics and patterns (details in S2 File). The model captured seasonal trends as major drivers and performed well over the validation period. There was, however, high fluctuation during the Rabi season, and the model did not perform ideally in this period due to high percentage error.

#### Qualitative parameter assessment

Based on the criteria of [43], the qualitative parameter assessment process was used to assess the reliability of the spectrum of information used in the model. The information and factors controlling their reliability were scored out of three (details in S1 File). We assessed 101 types of information through this process and found 10 types of information scoring below the threshold reliability score of 40%, all of which were informed based on modeler intuition owing to a lack of quantitative data on non-Loop price dynamics, productivity investments (i.e., yield) and consumption demand elasticity.

### Scenario descriptions and model outcomes

The representation of the horticultural system of Jashore provides a virtual laboratory in which to analyze the future implications of different policy and intervention options. We designed different potential scenarios that may improve the delivery of fruit and vegetables to small markets while avoiding trade-offs through a series of GMB sessions and reference group discussions in Jashore. We classified 14 scenarios into four groups: baseline; internal; external; and combination. The rationale and form of the 14 future scenarios are detailed in Table 3.

**Table 3:** Scenarios driving the aggregation scheme to explore the availability and affordability of F&V in the local consumer market considering farmer-facing trade-offs.

Group	Scenario name	Description and behavior in the model
1. Baseline	Counterfactual	What if aggregation stopped evolving? The number of farmers participating in aggregation does not change from October 2018 to December 2023.
	Baseline	What if aggregation only evolves naturally? There are no extension services, but adoption remains linked to the profitability of the scheme spreading between farmers via word of mouth.
2. Internal	Scaling-up Loop (S1)	Scaling up the aggregation on top of baseline via active extension services and word of mouth. We set the extension effectiveness value to 0.54 (farmers/non-Loop farmers/day) from April 2017 to September 2019 and to 0.30 (farmers/non-Loop farmers/day) during October 2019 to December 2023. Historical value 0.54 produces historical evolution for farmers, thus experienced from dashboard data.
	Double scaling up Loop (S2)	We doubled the S1 extension effectiveness value after validation (see S1 extension effectiveness value).
	Supply quota (S3)	To counteract the tendency for Loop supplies to be sent to LLMs, this scenario assumes farmers are mandated to sell 30% of their produce in LCMs.
	Transport subsidy for the smaller market (S4)	Loop farmers receive a blanket BDT 0.25 per kg subsidy for transporting their produce to the LCM using Loop. This subsidy is given to encourage Loop farmers to go to smaller markets like LCMs.
3. External	Farmgate cold storage (S5)	As per the SGMB discussion, farmgate cold storage are increasingly becoming popular as these help farmers to minimize price shocks. Usually, these are only used for potatoes and tomatoes, but they are also becoming popular for other vegetables, with a maximum 21- day shelf life (based SGMB). The cold-storage capacity was assumed to be 20% of the overall production volume of the region.
	Reference price growth (S6)	Reflecting an exogenous increase in demand from current demand, the price beyond which consumers in retails market perceive F&V to be expensive is increased by 5% per year due to minimum wage rate increases in Bangladesh as well as increases in purchasing power.
4. Combination	Scaling up Loop, supply quotas, and transport subsidies for smaller markets (S1+S3+S4)	This combination of scenarios aims to increase the supply to smaller markets whilst offsetting the additional costs. This includes extension efforts by Loop continuing at the same level after October 2019 (i.e., akin to S1 above); farmers are being mandated to sell 30% of their produce to LCMs (i.e., akin to S3 above); and farmers being given a transport incentive of BDT 0.25 per kg for supplying to LCMs.
	Scaling up aggregation, supply quota, and reference price growth (S1+S3+S6)	This combination of scenarios also aims to increase 30% supply and 5% price growth per year whilst simultaneously increasing farmer’s benefits at LCMs. Scaling up aggregation will be continuing at the same level of extension effort from October 2019.
	Scaling up current aggregation, farmer-side cold storage, and reference price growth at 5% (S1+S5+S6)	This combination of scenarios comprises the same level of extension effort from October 2019 with being introduced farmer-side cold storage and 5% growth per year of the current price. Farmers will store F&V at cold storage in 21 days shelf life for a better price.
	Double scaling up current aggregation, farmer-side cold storage, and reference price growth at 5% (S2+S5+S6)	This combination of scenarios comprises a double level of extension effort from October 2019 from the current aggregation effort with being introduced farmer-side cold storage and 5% growth per year of the current price. Farmers will store F&V in farmgate cold storage for up to a maximum of 21 days shelf life for better price.
	Double scaling up Loop, supply quotas, and transport subsidies for smaller markets (S2+S3+S4)	This combination of scenarios aims to increase the supply to smaller markets whilst offsetting the additional costs. This includes Double extension efforts by Loop continuing after October 2019; farmers being mandated to sell 30% of their produce to LCMs, and farmers being given a transport incentive of BDT 0.25 per kg for supplying to LCMs as a LOOP intervention.
	30% supply quota, transport subsidy at LCM and reference price growth per year at 5% (S3+S4+S6)	This combination of scenarios also aims to increase by 30% more supply with protecting price reduces by 5% price growth per year whilst simultaneously increasing farmer’s benefit at LCMs. This scenario will simulate based on baseline scenario at a time.

First, the baseline scenario group includes a counterfactual to explore what might happen if the intervention had not been scaled up over time. For example, it might be possible to show that farmers are participating due to linking word of mouth and this would not have happened without this action. Second, the scenarios are combined with scaling-up, a supply quota, transport subsidy and external scenarios are combined with cold storage facilities at farmgate, and reference price growth. The external scenarios are designed as supply shifts that can be influenced by the drivers within or outside of the value chain such as institutional changes, consumer needs or technological changes or other unforeseen events that disrupted the supply chain of many businesses [46].

In addition, we consider internal scenarios for farmers direct participation and making the enabling environment cheaper to encourage farmers to participate. Subsidized farmgate cold storage and transport cost interventions can be paid by the DG and government and reduce gradually over time. On the other hand, quota systems can be beneficial tools to avail potential benefits to farmers [46]. Thus, Loop mandated to supply 30% (Supply quota) of F&V to smaller market to increase the availability for consumers. Finally, combined scenarios are considered to examine the interaction of improvement of F&V supply to neglected smaller market with different technical interventions [47]. So, whether combining individual scenarios has a disproportionate impact on the outcomes and whether multiple interventions can overcome any trade-offs are the main focuses of this study.

As the visualization of outcomes often becomes complicated when there are too many scenario comparisons, we only track the five following core outcomes:

1. Monthly average price of vegetables in LCMs (Tk/kg) (the main indicator of vegetable affordability).
2. Monthly average per consumer purchases (kg) (dependent on availability and price).
3. Monthly average profit per Loop farmer per unit (Tk) (weighted average of Loop farmer F&V profit per farmer per unit (Tk/kg) from sales in the three market types).
4. Monthly average profit per farmer per unit (Tk) including Loop and non-Loop- farmers (weighted average of all farmer profits per unit (Tk/kg)).
5. Monthly average total vegetables supply from Loop farmers (kg)

**Fig 3:**
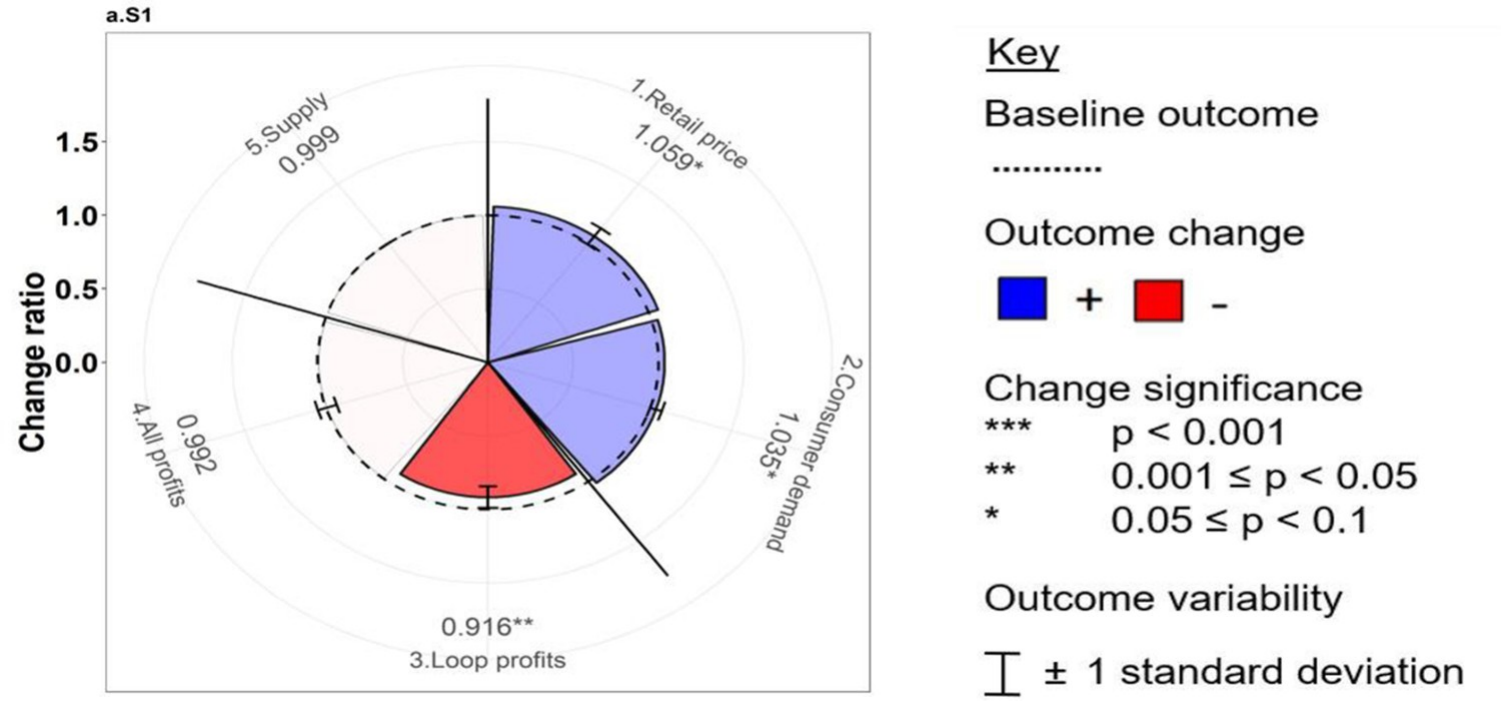
Schematic example of the trade-off wheel comparing the change ratios of the five outcomes (1–5) under the hypothetical future scenario “S1” relative to their respective baseline values. Note: The price outcome is inverted (i.e., positive change represents cheaper vegetables).

We developed novel trade-off wheels to aggregate the monthly output time series (S4 File) and analyze the direction, magnitude, and significance of changes in each outcome (Fig 3) [48]. The above six outcomes populate the radial x-axis, with the mean change ratio of each outcome (*i*) under each scenario (*j*) calculated as follows:

$${Change\;ratio}_{ij}=mean(\frac{{New\;value}_{ijt}}{{Baseline\;value}_{ijt}})$$ (1)

where t represents the simulation month.

Fig 3 shows that of the five outcomes associated with the hypothetical simulation, significant positive changes only occur in monthly retail prices at LCMs and that, simultaneously, consumer purchases increase. However, the Loop profit of participating farmers is significantly reduced relative to the baseline. Finally, two sample t-tests were performed to determine whether the outcome values under each intervention were significantly different from the baseline.

## Results

### Consequences of the current Loop aggregation scheme

We first visualize the dynamics associated with the baseline evolution of aggregation adoption relative to the counterfactual scenario, which holds aggregation participation constant at 16% of the total farmer population based on Loop dashboard data. We then analyze the alternative scenarios to improve the equitable distribution of vegetables at LCMs.

In comparison to the counterfactual scenario, the volume of F&V aggregated in the final year of the baseline scenario is 2.5 times greater than in the counterfactual (S3 File). This is because 4,780 farmers benefit from lower transport costs, better market access, and higher relative market prices from the current Loop aggregation scheme. However, by increasing participation as well as aggregation, farmers are more likely to transport their vegetables to LLMs due to the relative guarantee of sale and the availability of a relatively large number of traders. The baseline relative to counterfactual does not produce significant differences in monthly consumer purchases or price.

We then visualized the current aggregation scheme using counterfactual and baseline scenarios, and the findings suggest that there are few benefits for consumers or producers under the baseline scenario relative to holding participation constant. Therefore, alternative future scenarios were tested to understand how the scheme can drive the horticultural system towards more equitable distribution whilst retaining farmer benefits (See S4 File).

### Potential market interventions and their trade-offs

**Fig 4:**
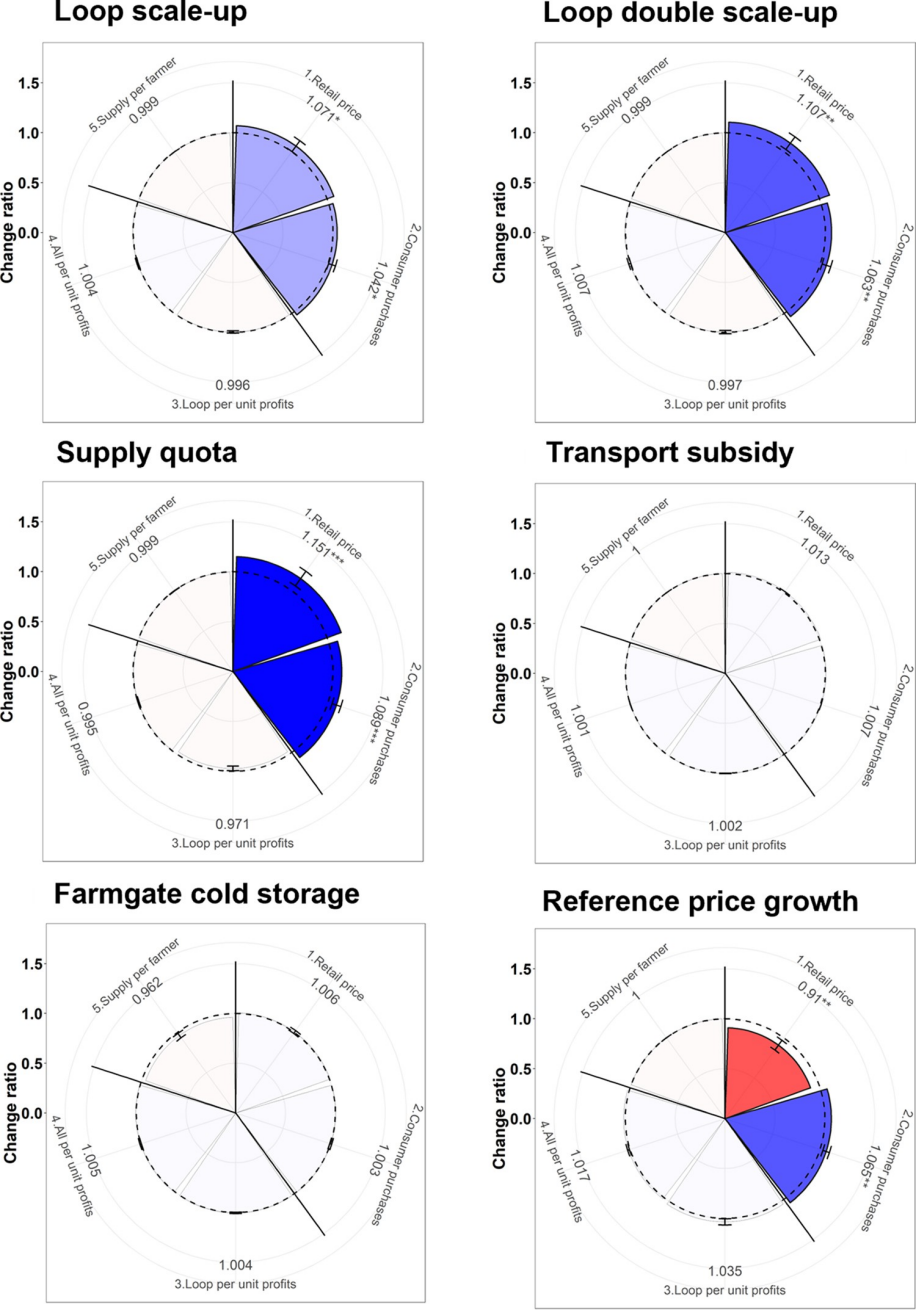
Trade-off wheels showing the results of different scenarios: (Loop scale-up) scaling-up of aggregation; (Loop double scale-up) double scaling-up of aggregation (Supply quota) 30% mandated supply to LCMs; (Transport subsidy) 50% Loop transport subsidies for LCM markets; (Farmgate cold storage) farmer-side cold storage and (Reference price growth) 5% reference price growth. Note: See S4 File for more details of the time series underlying these plots.

In this section, we explain how different market interventions generate outcomes associated with six individual scenarios (Fig 4). Improving the availability and affordability of vegetables in LCMs is largely dependent on significant improvement in both distribution and the market environment.

Fig 4 (Loop scale-up) shows that scaling up Loop from 4,780 to 15,500 Loop farmers over six years relative to the baseline simulation is associated with a decreased mean monthly market price in LCM. Whilst the actual percentage of Loop produce going to LCMs decreases, owing to Loop farmers having better access to the LLM, the tripling in Loop farmer numbers leads to a 3.5% increase in total F&V supplies from Loop farmers to LCM. In turn, the resulting decrease of 4.5% in monthly average retail prices results in a monthly average consumer purchase increase of 3%. On the other hand, the monthly Loop profit decreases by 6%, but total farmer profits due to price increases in each market and supply per farmer volume remain the same. Doubling the scaling-up rate [Fig 4 (Loop double scale-up)] causes participation in the aggregation scheme to increase from 4,780 to 20,500 relative to baseline simulation. As the overall number of Loop farmers increases, the monthly market price decreases at LCMs, LSMs, and LLMs. The actual percentage of Loop produce going to LCMs decreased but the total number of farmers increased. At the same time, the proportion of Loop produce going to LSMs increases but remains the same for LLMs, although the total number of farmers increases. This means that farmers joining the Loop have better access to larger markets. Monthly average retail prices significantly decreased by 7% due to market supply increases of 5.69%, leading to the average monthly consumer purchases increase by 4.40%. Simultaneously, Loop farmer profits decreased significantly by 10.7% due to market price going down compared to the baseline scenario but, overall, both Loop and non-Loop farmers’ profits and supply per farmer changed, resulting in insignificant.

Scaling up aggregation can lead to improvements in F&V availability in local consumer markets because although fewer Loop farmers go to LCM as a *proportion* of all Loop farmers (e.g., 6% rather than 11% in the baseline), the *absolute number* of Loop farmers going to LCM increases by 6% (owing the scaling of aggregation and the saturation of the large local market). This suggests that scaling up aggregation across a horticultural value chain such as Jessore can simultaneously enable a) a greater proportion of farmers to reach higher demand markets, and b) enable more farmers to supply LCM (but without negative consequences on revenues and profits i.e., perhaps due to lower transport costs as a result of aggregation participation).

The supply quota [Fig 4 (Supply quota)] scenario mandates a minimum of 30% of total aggregation to LCMs to improve availability and affordability. The remaining 70% of the aggregation is distributed to LSMs and LLMs based on the market preferences of farmers. The supply quota increases farmer participation by 8% in LCMs but the supply of produce from Loop farmers to LSMs is shifted mostly to LLMs as better prices are available. Thus, the monthly average retail price decreases by 12.5% at LCMs while monthly average consumer purchases increase by 6.4%. Direct small market quotas may be an effective strategy to increase the availability and affordability of LCMs. To avoid a significant negative trade-off of weaker prices and lower capacities in LCM, the model suggests that the farmers not involved in the quota unanimously opt to supply to LLMs over the local small wholesale market (LSM).

The reference price growth [(Fig 4 (Reference price growth)] scenario increases the price beyond which consumers perceive F&V as unaffordable or expensive by 5%. The exogenous causes, such as seasonal variability and food habit changes, are due to increasing national minimum wages. It increases consumer purchases monthly by 5.31% through monthly price increases as national purchasing power increases.

Simultaneously, no categories (Loop and non-Loop) were revealed in which farmers experience profit or losses, owing to LLMs providing better prices. Subsequently, the transport subsidies (50%) for LCMs [Fig 4 (Transport subsidy] and cold storage [Fig 4 (Farmgate cold storage)] scenarios do not produce significantly different outcomes from the baseline scenario. This is because the lower total transport costs (4% of total input cost) based on Loop farmer participation at LCMs increased by 1.5%. Still, Loop farmer participation in LSMs and LLMs slightly decreased. Farmgate cold storage also encourages Loop farmers to participate in LCMs but not significantly.

All individual interventions/scenarios can play key roles in making Loop and Loop-like aggregation services more sensitive to the demands of consumers dependent upon local retail-oriented markets. All interventions have no significant (i.e., transport subsidy and farmer-side cold storage) impact on all of the five outcomes simultaneously, but some of them generate significant changes in outcomes. Notably, the scaling of aggregation is a key casual dynamic in relation to the fair distribution of vegetables and leads to better access to larger markets. Further, the supply quota mandating 30% of total Loop aggregation going to LCMs increases availability and affordability without harming farmers’ profits. In the next section, we will investigate how combinations of interventions can change the outcomes significantly.

### Combination of intervention and emerging multi-dimensional trade-offs

**Fig 5:**
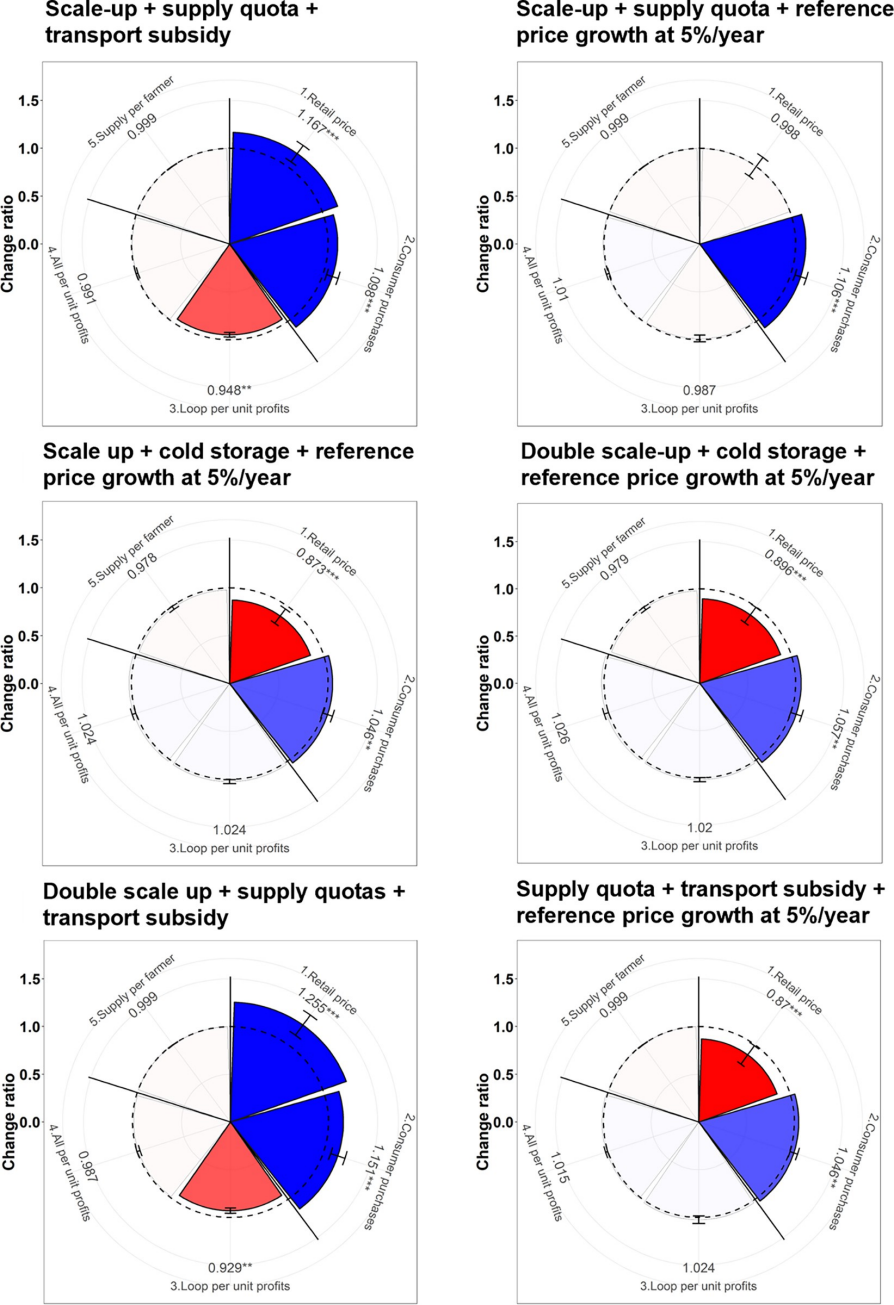
Trade-off wheels resulting from the six combination scenarios (see details in Table 2). Note: See S4 File for the time-series data underlying these plots.

Fig 5 shows the combination of various interventions and the resulting trade-off between farmers and consumer purchases for F&V. We first combine the 30% supply quota and transport subsidies scenario for LCMs with the scaling up of aggregation [Fig 5 (Scale-up+Supply qouta+Transport subsidy)]; as a result, the actual proportion of Loop farmers increases at LCMs while the rest of the Loop farmers choose their preferred market based on price. This finding shows that the rest of the Loop farmers are not interested in LSMs but instead go to LLMs due to relatively higher prices, although the total amount of supply decreases.

The monthly average retail price of vegetables decreases by 32% as the supply increases by 23% due to farmer participation increasing by 80%, leading to monthly average consumer demands increasing by 16.5%. This combination shows that changes in the monthly average retail price and consumer purchases are highly significant. On the other hand, this combined scenario is resulting in saturation, which reduces the monthly average Loop farmer profits by 7.7% as profits directly depend on price relative to baseline, while non-Loop farmer profits remain unchanged.

Second, we combine the scaling up the aggregation with 30% quota and reference price growth [Fig 5 (Scale-up+ Supply quota+ Reference price growth at 5%/year)]. The actual proportion of Loop farmers increases at LCM due to the inclusion of a fixed quota but decreases in LSM and increases in LLM. The participation of Loop farmers is determined by the market preference based on price. Loop farmers are less interested in LCM as lower price than LLM. This result shows that monthly average retail price does not change significantly but consumer purchases increase significantly by 7.5% due to total supply increases in LCM by 12%. Therefore, Loop and non-Loop monthly weighted profits do not change significantly.

Third and fourth, we combine the scaling up and double scaling up with farmer-side cold storage and reference price growth at 5% [Fig 5. (Scale-up+ Cold storage+ Reference price growth at 5%/year)] and [Fig 5. (Double scale-up+Cold storage+ Reference price growth at 5%/year)] to look at consumer purchases and farmer’s benefits. Total as well as Loop farmers participation increase due to relative higher price. On the other hand, the proportion of Loop farmers slightly decreased but the price is lower compared to the baseline. Overall, the price is better, and farmers participation are increased than above combination scenarios but still, farmers are supplying more to LLM as offering higher price relative to other market. This is a fact that LLM has the highest number of traders with the highest purchase quantity of F&V. In addition, a large market like LLM is well connected with distant traders and along with sales guarantee. The findings also show that the monthly average retail price increased significantly by 11.50% and 7.50%, respectively and consumer purchases increased by 2.50% and 4%, respectively due to increases in the reference price growth at 5%. We have also seen in RVCA that the decreases in price are due to a high seasonal supply of F&V and an increase in the purchasing power of consumers.

Fifth, this scenario is comprised of the 30% supply quota and transport subsidies scenario with the scaling up of aggregation [Fig 5 (Double scale-up+ Supply quota+ Transport subsidy)] targeting the LCM for availability. As a result, the actual proportion of Loop farmers increases at LCMs while the rest of the Loop farmers choose their preferred market based on price. This finding shows that the rest of the Loop farmers are not interested in LSMs but instead going to LLMs due to relatively higher prices, although the total amount of supply decreases. The monthly average retail price of vegetables decreased by 19% as the supply increased by 16% due to farmer participation increasing by 136%, leading to monthly average consumer demands increasing by 10%. This combination shows that changes in the monthly average retail price and consumer purchases are highly significant. On the other hand, this combined scenario is resulting in saturation which reduces the monthly average Loop farmer profits by 5%, as profits directly depend on price relative to baseline, while non-Loop farmer profits remain unchanged.

Sixth, the final combination comprises a 30% quota with the transport subsidy and reference price growth focusing on LCM [Fig 5 (Supply quota+ Transport subsidy+ Reference price growth at 5%/year)]. Total as well as Loop farmers participation increased due to relatively higher price. On the other hand, the proportion of Loop farmers slightly decreased but the price is lower compared to baseline. Overall, the price is better, and participation improved than the above combination scenarios but still, farmers are supplying more to LLM as offering higher prices relative to other market. The findings also show that the monthly average retail price increased significantly by 5.65% and consumer purchases increased by 2.50% although increases the reference price growth at 5% per year. Both Loop and non-Loop profits get also change but it is positively insignificant.

## Discussion

To achieve mutual benefits in future, decision makers need to consider the power of aggregation schemes to influence horticultural supply dynamics [42], as well as the diverse range of actors involved within the wider food system [49]. We have developed a system dynamic modeling framework to identify the future internal and external scenarios that harness the equitable distribution of vegetables to nutrition vulnerable areas while avoiding trade-offs. Considering these contexts, we discuss in this section: policy implications/implications for aggregation; extension and limitation of the model; and future research directions.

### Policy implications/implications for aggregations

Despite the importance of nutrition for local people in developing countries, policies enacted by national governments often have shortcomings in terms of improving nutritional outcomes through a market-driven approach [50]. A fragmented market system and high transportation costs make vegetable consumption costlier for price-sensitive consumers. This also works against farmers as they often lose revenue due to unfavorable market dynamics. Hence, the National Agriculture Policy (NAP) of Bangladesh has emphasized more efficient value chain linkage and well-functioning markets. The DAE’s Agriculture Extension Manual and the National Agriculture Extension Policy (NAEP) also emphasize community-led market approaches but do not provide a clear directive on how to implement a community-led approach.

Findings from our study illustrate potential scenarios to increase income and profits based on the farmer-led vegetable aggregation model. Community-level aggregation helps farmers to obtain better negotiating power, thus increasing their profitability. This provides policymakers with a clear-pathway and a model to help successfully implement the NAP and NAEP.

However, our study demonstrates that given the wider costs involved in agricultural marketing and transportation, such subsidies and different interventions may have a limited impact on farmer profits and consumer demand. This is also supported by [51] study, which showed that blanket government subsidies often do not bring significant benefits. Therefore, moving away from a blanket subsidy policy approach, a more targeted pro-poor intervention targeting smallholder farmers targeting LCMs should be considered.

The current Bangladeshi agricultural output market is governed by the Bangladesh Agriculture Marketing Ordinance 2018, which focuses on ensuring a fair price for farmers by imposing various safeguarding regulations. However, this policy does not provide any protection for the needs of consumers. To this end, our study demonstrates that the most significant driver for improving vegetable consumption among local consumers is ensuring that a certain volume of vegetables always goes to LCMs. In our model, we simulated a mandatory continuous flow of 30% aggregated vegetable flow to such small markets. The findings from our study show that, even after enforcing this market constraint there is no loss of profit for farmers. This is because farmers are not constrained in their market supply i.e., the remaining 70% of aggregating farmers opt to supply LLMs (with better prices and greater capacity) over LSMs. This implies that to improve nutritional outcomes focus should be given to introducing safeguarding interventions for local consumers by ensuring a continuous flow of produce to LCMs. However, implementing any market policy that aims to govern the flow of agricultural produce to specific markets has always been challenging [52]. Hence, to motivate farmers, a multi-pronged approach encompassing, for example creating awareness, the introduction of transport subsidies for smallholder farmers, or current price growth per year to ensure the continuous flow of agricultural produce to LCMs.

Our study explored how a fixed quota system influences the availability and affordability of F&V for local consumers at LCMs without undermining farmer benefits. It is observed that the introduction of a quota is economically thriving and profitable for neglected community fisheries, makes the eco-system sustainable[53], and achieving equitable changes[54]. In addition, under the enforced quota system, quota markets can obtain several implications for the current and future equilibrium [55].

The key policy implications from our simulation should be to ensure a safeguarding mechanism for local consumers living in rural communities. A new market governance approach is needed by NAMO-2018 based on ensuring a continuous flow of F&Vs to LCMs. Moving away from blanket input subsidies, more intervention to motivate farmers to sell at LCMs will create a constant flow of F&Vs to LCMs, which can significantly improve the consumption of F&V among rural consumers. Our study has explored some innovative pathways for aggregation to create a win-win environment both for farmers and consumers-facing local small market demand.

### Extensions and limitations

The transferability of a model is an important aspect of any scientific discovery. Whilst decision-makers often rely on high accuracy forecasts, each exploratory element of the model can be used to investigate the due diligence and trade-offs within the system path. Where quantitative data and computer-aided tools are limited, a casual diagram can be developed to identify the outcomes by investigating potential due diligence and trade-offs evolving from the divergent futures. Spatial and temporal data can also be used to improve the outcomes resulting from modeling practices.

This model represents a first attempt to identify scenarios within the Jashore vegetable value chain that overcome trade-offs between producers and consumers. However, we appreciate that our model, like all models, has uncertainties, including: i) the averages and aggregation we have made, between different farmer types (e.g., marginal, and non-marginal), different vegetable items, and their value chains. As an illustration, the land size, type and economic value (i.e., price) of vegetables supplied by different farmers will vary in reality, and whilst beyond the scope of the current study, factoring in such differences may be a direction for future research using sensitivity analysis-based simulations approaches and/or weighted sampling survey strategies [56, 57] ii) the lack of spatial dimensions within the model, as common amongst quantitative system dynamics approaches; and iii) the lack of non-Loop timeseries data to help parameterize the non-Loop interactions. iii) Primary surveys both quantitative and qualitative are based on recall-based questions that often lead to measurement errors [58, 59]. The study was designed to conduct a survey in a way such as focused on short period question so that respondents can remember easily and be ready to answer.

In addition, the variables found to be critically sensitive during the qualitative parameter assessment also require further research to improve the accuracy of the model. Therefore, to help improve confidence in these particular variables, further research may focus on the different rates at which farmers in the local context invest in more land and yield-improving technologies and, critically, whether these vary between seasons. Directions for future research efforts could involve longer time horizons and adding additional drivers of food systems, such as consumer behavior, population trends, climate vulnerability, environmental factors, and food safety indicators [60]. In addition, supplementary drivers of food choice/attributes, such as taste, appearance, quality, freshness, and extra internal issues, such as food wastage versus external issues, such as price shocks and cold storage, represent further directions for future research efforts.

## Conclusions

This study was designed based on the Loop aggregation scheme in the Jashore district in Bangladesh. We developed a novel modeling framework based on multidimensional datasets to identify the value chain trade-offs considering farmers’ benefits and improving supply to consumer-facing local retail markets. The study investigated multiple scenarios based on the Loop aggregation scheme to assess how the scheme can improve equitable vegetable distribution to local consumer-facing markets, whilst overcoming value chain trade-offs and multi-dimensional challenges in the food system.

Increasing aggregation to local markets has a positive impact on increasing demand in rural consumer markets, but there are trade-offs for participating farmers and their profits. Only a 30% fixed quota and reference price growth per year at 5% for local retail markets produces win -win trade-off, thus increasing consumer purchases whilst avoiding farm losses. Fixed quota is mandated and achieved the equitable F&V distribution among rural and urban markets. It is also established that the quota system has many implications for current and future market equilibrium. We also see higher customer purchases, as customers are now able to purchase more at a given price (e.g., customers become more resilient to seasonally higher prices etc.). Therefore, these results suggest that a fixed quota system that can increase supplies and increase demands in local consumer markets would lead to a ’win-win’. This study’s aim is not to evaluate all possible value chain trade-offs, but rather to investigate new interventions and new packages to increase F&V availability and affordability in the context of developing countries.

## Supporting information

S1 File Reliability test—qualitative parameter assessment. (DOCX)

S2 File Reliability test—behavior reproduction. (DOCX)

S3 File Current Loop aggregation scheme. (DOCX)

S4 File Scenarios results under the once-at-a-time (OAT) and combo. (DOCX)

S5 File Model equations and parameter values. (DOCX)
