# Supplementary material for: Identifying value chain trade-offs from fruit and vegetable aggregation services in Bangladesh using a system dynamics approach
Source: PLoS One. 2024 Jan 24;19(1):e0297509. doi: 10.1371/journal.pone.0297509 (PMC10807782; doi:10.1371/journal.pone.0297509)
Supplement: S1 File — (DOCX) [file pone.0297509.s001.docx]

**Supporting information 1: Reliability test - qualitative parameter assessment**

[1] developed a qualitative assessment to assign a reliability score to every model parameter, whereby the spatial applicability and transferability of the underlying data, plus the quantity of evidence and any statistical confidence, are each scored out of three (giving a total of 12). The assessment identifies the most qualitatively uncertain variables as those scoring below the threshold (the mean score minus the standard deviation).

Of the 101 parameters assessed, 14 (14%) scored beneath the reliability threshold (S1 Table). All unreliable parameters were informed by modeler intuition in the absence of a reliable survey, Loop dashboard, or SGMB data.

S1 Table: Output table of the qualitative parameter assessment for the Jashore system dynamics model – based on the methodology of [1]. The mean average reliability score equals 67.33% (n = 101), and the standard deviation equals 14.22%. The reliability threshold score equals 53.10%, and variables below the threshold are highlighted in red.

| **Module** | **Variable** | **Sources** | **Information type** | **Score (%)** |
| --- | --- | --- | --- | --- |
| Farmer | Extension effectiveness | LOOP Dashboard | Statistical information | 58.33 |
|  | Word of mouth effectiveness | LOOP Dashboard | Statistical information | 58.33 |
|  | LOOP drop out | LOOP Dashboard | Statistical information | 66.67 |
|  | Population in 30 villages | National data | Statistical information | 75.00 |
|  | Death rate | National data | Statistical information | 75.00 |
|  | Birth rate | National data | Statistical information | 75.00 |
| Land | Land holding during Rabi | SGMB | Expert stakeholder knowledge | 83.33 |
|  | Land holding during Kharif-1 | SGMB | Expert stakeholder knowledge | 83.33 |
|  | Land holding during Kharif-2 | SGMB | Expert stakeholder knowledge | 83.33 |
|  | Total vegetables land stock | MINI survey data | Statistical information | 58.33 |
| Production & Aggregation | Rabi yield | DAE data | Statistical information | 66.67 |
|  | Khairf-1 yield | DAE data | Statistical information | 66.67 |
|  | Kharif-2 yield | DAE data | Statistical information | 66.67 |
|  | Rabi supply frequency | SGMB | Expert stakeholder knowledge | 83.33 |
|  | Kahrif-1 supply frequency | SGMB | Expert stakeholder knowledge | 83.33 |
|  | Kharif-2 supply frequency | SGMB | Expert stakeholder knowledge | 83.33 |
|  | LOOP vegetables eaten per HH | MINI survey data | Statistical information | 75.00 |
|  | LOOP vegetables given away per HH | MINI survey data | Statistical information | 75.00 |
|  | NL vegetables eaten per HH | MINI survey data | Statistical information | 75.00 |
|  | NL vegetables given per HH | MINI survey data | Statistical information | 75.00 |
|  | Aggregation per week | LOOP Dashboard | Statistical information | 58.33 |
|  | Farmers per aggregators | LOOP Dashboard | Statistical information | 58.33 |
|  | Aggregator’s capacity | LOOP Dashboard | Statistical information | 58.33 |
|  | LOOP input cost (Per day) | MINI survey data | Statistical information | 75.00 |
|  | LOOP labor cost (Per day) | MINI survey data | Statistical information | 75.00 |
|  | NL input cost (Per day) | MINI survey data | Statistical information | 75.00 |
|  | NL labor cost (Per day) | MINI survey data | Statistical information | 75.00 |
| Market aggregation | LLM proportion NL | SGMB | Expert stakeholder knowledge | 66.67 |
|  | LLM proportion LOOP | LOOP Dashboard | Statistical information | 58.33 |
|  | LLM trader's capacity | SGMB | Expert stakeholder knowledge | 83.33 |
|  | Number of traders at LLM | SGMB | Expert stakeholder knowledge | 66.67 |
|  | LSM proportion NL | SGMB | Expert stakeholder knowledge | 66.67 |
|  | LSM proportion LOOP | LOOP Dashboard | Statistical information | 58.33 |
|  | LSM trader's capacity | SGMB | Expert stakeholder knowledge | 66.67 |
|  | Number of traders at LSM | SGMB | Expert stakeholder knowledge | 66.67 |
|  | LCM proportion NL | SGMB | Expert stakeholder knowledge | 66.67 |
|  | LCM proportion LOOP | LOOP Dashboard | Statistical information | 58.33 |
|  | LCM trader capacity | SGMB | Expert stakeholder knowledge | 66.67 |
|  | Number of retailers at LCM | SGMB | Expert stakeholder knowledge | 66.67 |
| Price | Actual sales price at LLM | LOOP Dashboard | Statistical information | 66.67 |
|  | Coverage perception time LLM | Modeller intuition’ | Statistical information | 75.00 |
|  | Sensitivity of Price to Inventory coverage LLM | Modeller intuition’ | Statistical information | 75.00 |
|  | Sensitivity of Price cost LLM | Modeller intuition’ | Statistical information | 75.00 |
|  | Market cost LLM (Taka/Kg) | SGMB | Expert stakeholder knowledge | 83.33 |
|  | Labor cost LLM (Taka/Kg) | SGMB | Expert stakeholder knowledge | 83.33 |
|  | Wastage cost LLM (Taka/Kg) | SGMB | Expert stakeholder knowledge | 66.67 |
|  | Vegetable’s cost LLM (Taka/Kg) | SGMB | Expert stakeholder knowledge | 66.67 |
|  | Actual sales price at LSM | LOOP Dashboard | Statistical information | 66.67 |
|  | Coverage perception time LSM | Modeller intuition’ | Statistical information | 75.00 |
|  | Sensitivity of Price to Inventory Coverage LSM | Modeller intuition’ | Statistical information | 75.00 |
|  | Sensitivity of Price cost LSM | Modeller intuition’ | Statistical information | 75.00 |
|  | Market cost LSM (Taka/Kg) | SGMB | Expert stakeholder knowledge | 83.33 |
|  | Labor cost LSM (Taka/Kg) | SGMB | Expert stakeholder knowledge | 66.67 |
|  | Wastage cost LSM (Taka/Kg) | SGMB | Expert stakeholder knowledge | 66.67 |
|  | Vegetable’s cost LSM (Taka/Kg) | SGMB | Expert stakeholder knowledge | 83.33 |
|  | Actual sales price at LCM | LOOP Dashboard | Statistical information | 66.67 |
|  | Coverage perception time LCM | Modeller intuition’ | Statistical information | 33.33 |
|  | Sensitivity of Price to Inventory Coverage LCM | Modeller intuition’ | Statistical information | 33.33 |
|  | Sensitivity of Price cost LCM | Modeller intuition’ | Statistical information | 33.33 |
|  | Market cost LCM (Taka/Kg) | SGMB | Expert stakeholder knowledge | 83.33 |
|  | Labor cost LCM (Taka/Kg) | SGMB | Expert stakeholder knowledge | 66.67 |
|  | Wastage cost LCM (Taka/Kg) | SGMB | Expert stakeholder knowledge | 66.67 |
|  | Vegetables cost LCM (Taka/Kg) | SGMB | Expert stakeholder knowledge | 83.33 |
| LOOP market preference | Market price time | SGMB | Expert stakeholder knowledge | 66.67 |
|  | Market trust time | SGMB | Expert stakeholder knowledge | 66.67 |
|  | Transportation cost (Taka/kg) | LOOP Dashboard | Statistical information | 66.67 |
|  | Tola (%) | SGMB | Expert stakeholder knowledge | 83.33 |
|  | Dholta (%) | SGMB | Expert stakeholder knowledge | 83.33 |
| Transport (Truck-Pickup) | Vehicle capacity | SGMB | Statistical information | 58.33 |
|  | Ferry cost | SGMB | Expert stakeholder knowledge | 83.33 |
|  | Toll fee | SGMB | Expert stakeholder knowledge | 83.33 |
|  | Profit per trip | SGMB | Expert stakeholder knowledge | 66.67 |
|  | Fuel economy | SGMB | Statistical information | 58.33 |
|  | Market distance covered | SGMB | Expert stakeholder knowledge | 83.33 |
|  | Fuel cost | SGMB | Expert stakeholder knowledge | 83.33 |
|  | Monthly maintenance cost | SGMB | Expert stakeholder knowledge | 83.33 |
|  | Vehicle price | SGMB | Statistical information | 58.33 |
|  | Vehicle lifetime | SGMB | Statistical information | 58.33 |
| Transport (Alamshadhu/Nachiman) | Capacity | SGMB | Statistical information | 58.33 |
|  | Per trip profit | SGMB | Expert stakeholder knowledge | 66.67 |
|  | Diesel cost | SGMB | Expert stakeholder knowledge | 83.33 |
|  | Distance covered | SGMB | Expert stakeholder knowledge | 83.33 |
|  | Maintenance cost | SGMB | Expert stakeholder knowledge | 66.67 |
|  | Vehicle price | SGMB | Statistical information | 58.33 |
|  | Vehicle lifetime | SGMB | Statistical information | 58.33 |
|  | Driver cost | SGMB | Expert stakeholder knowledge | 83.33 |
| LOOP Trust | Trust perception time | SGMB | Expert stakeholder knowledge | 66.67 |
|  | Difference profit weight | SGMB | Expert stakeholder knowledge | 66.67 |
| Yield investment LOOP | LOOP yield improvement | SGMB | Expert stakeholder knowledge | 66.67 |
|  | Yield investment rate | Personal intuition | Modeler intuition | 33.33 |
|  | LOOP land investment rate | SGMB | Expert stakeholder knowledge | 66.67 |
| Yield investment NL | Non LOOP yield improvement | Personal intuition | Expert stakeholder knowledge | 66.67 |
|  | Yield investment rate | Personal intuition | Modeler intuition | 33.33 |
|  | Non-LOOP land investment rate | SGMB | Expert stakeholder knowledge | 66.67 |
| Consumption | Average household member | MINI survey data | Statistical information | 66.67 |
|  | Maximum consumption | Personal intuition | Modeler intuition | 33.33 |
|  | Reference consumer purchases | Personal intuition | Modeler intuition | 33.33 |
|  | Demand elasticity | Personal intuition | Modeler intuition | 33.33 |
|  | Reference price | Personal intuition | Modeler intuition | 33.33 |
|  | Demand adjustment delay | Personal intuition | Modeler intuition | 33.33 |
|  | Initial customer per retailer | Personal intuition | Modeler intuition | 33.33 |
|  | Consumer growth rate | Personal intuition | Modeler intuition | 33.33 |
|  | Customer rout in | SGMB | Expert stakeholder knowledge | 50.00 |

# Supplementary reference

1. Chapman, A., Darby, S., 2016. Evaluating sustainable adaptation strategies for vulnerable mega-deltas using system dynamics modelling: Rice agriculture in the Mekong Delta’s An Giang Province, Vietnam. Science of The Total Environment 559, 326–338. <https://doi.org/https://doi.org/10.1016/j.scitotenv.2016.02.162>
