# Supplementary material for: Identifying value chain trade-offs from fruit and vegetable aggregation services in Bangladesh using a system dynamics approach
Source: PLoS One. 2024 Jan 24;19(1):e0297509. doi: 10.1371/journal.pone.0297509 (PMC10807782; doi:10.1371/journal.pone.0297509)
Supplement: S2 File — (DOCX) [file pone.0297509.s002.docx]

**Supporting information 2: Reliability test - behavior reproduction**

S1 Fig: Timeseries comparing modelled outputs to Loop dashboard data of the number of Loop farmers in Jashore district.

S2 Fig: Time-series comparing modelled outputs to Loop dashboard data of daily supply of Loop farmers in Jashore district

S3 Fig: Timeseries comparing modelled outputs to Loop dashboard data of daily Loop aggregation at Local Large Market (LLM) in Jashore district.

S4 Fig: Timeseries comparing modelled outputs to Loop dashboard data of daily total Loop aggregation at all market in Jashore district.

**S2 Table:** Characteristics and performance metrics of important modeled and observed data drivers and outputs (PE-Percentage error)

| Variables | Unit | Observed mean | Modeled mean | PE % |
| --- | --- | --- | --- | --- |
| Loop Farmers | Number | 2130.476 | 1847.997 | 13.25896 |
| Daily aggregation | Kg | 26382.43 | 34218.7 | -29.7026 |
| Daily supplied Loop farmer | Number | 133.4213 | 184.0242 | -37.9272 |
| LLM Supply | KG | 20514.74 | 21944.4 | -6.96894 |
