# Supplementary material for: Identifying value chain trade-offs from fruit and vegetable aggregation services in Bangladesh using a system dynamics approach
Source: PLoS One. 2024 Jan 24;19(1):e0297509. doi: 10.1371/journal.pone.0297509 (PMC10807782; doi:10.1371/journal.pone.0297509)
Supplement: S3 File — (DOCX) [file pone.0297509.s003.docx]

**Supporting information 3: Current Loop aggregation scheme**


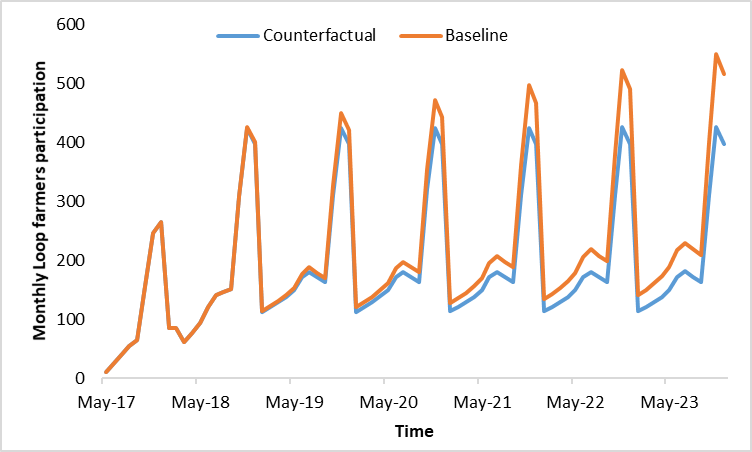

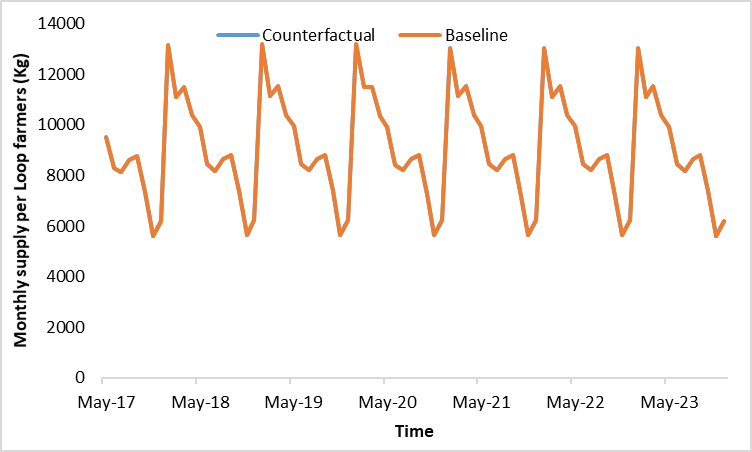

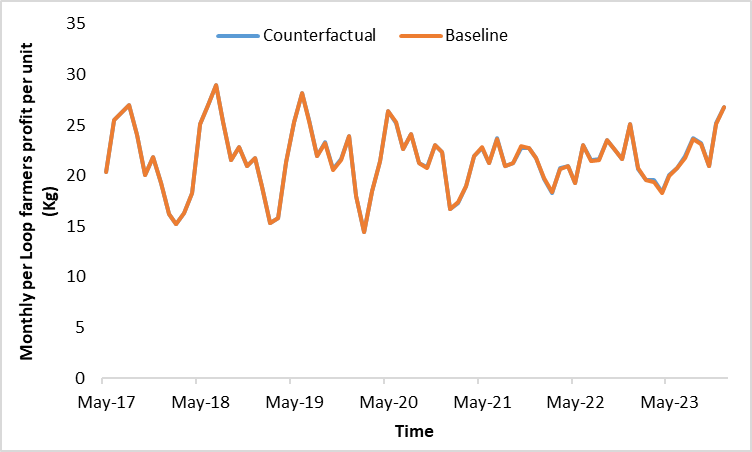

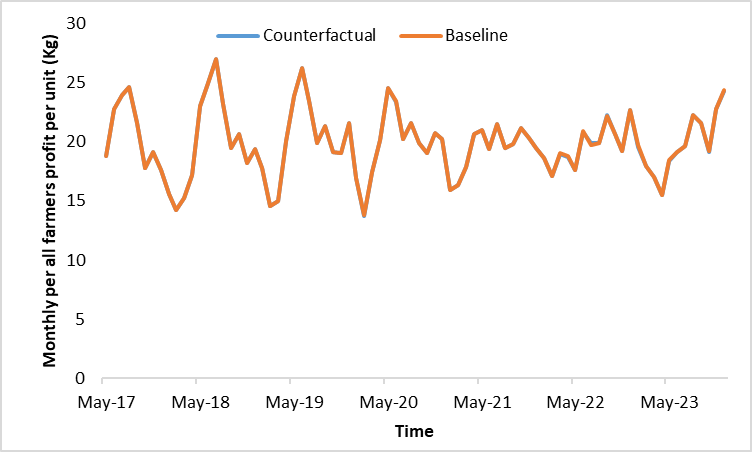

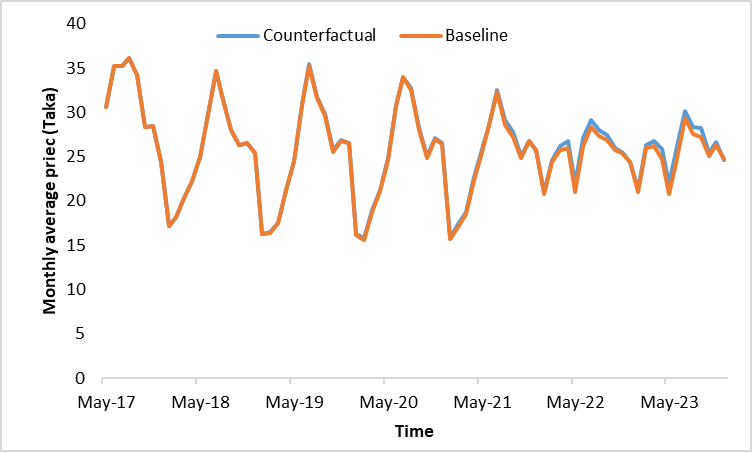

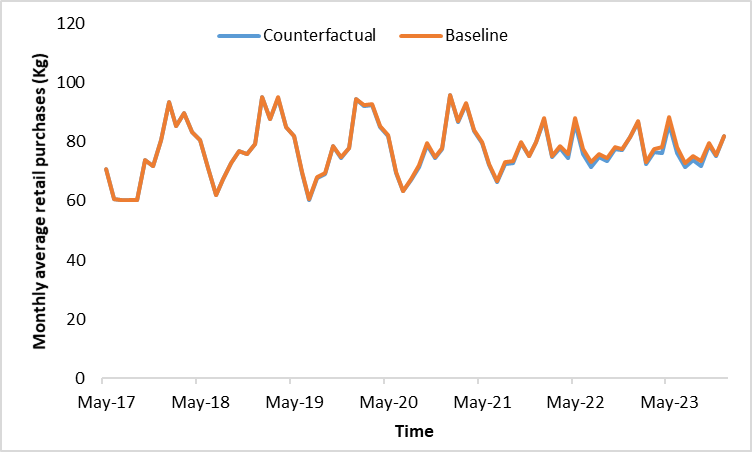


S5 Fig: Timeseries comparison of the ‘baseline’ and ‘counterfactual’ scenarios.
