# Supplementary material for: Identifying value chain trade-offs from fruit and vegetable aggregation services in Bangladesh using a system dynamics approach
Source: PLoS One. 2024 Jan 24;19(1):e0297509. doi: 10.1371/journal.pone.0297509 (PMC10807782; doi:10.1371/journal.pone.0297509)
Supplement: S4 File — (DOCX) [file pone.0297509.s004.docx]

**Supporting information 4: Scenarios results under the once-at-a-time (OAT) and combo.**


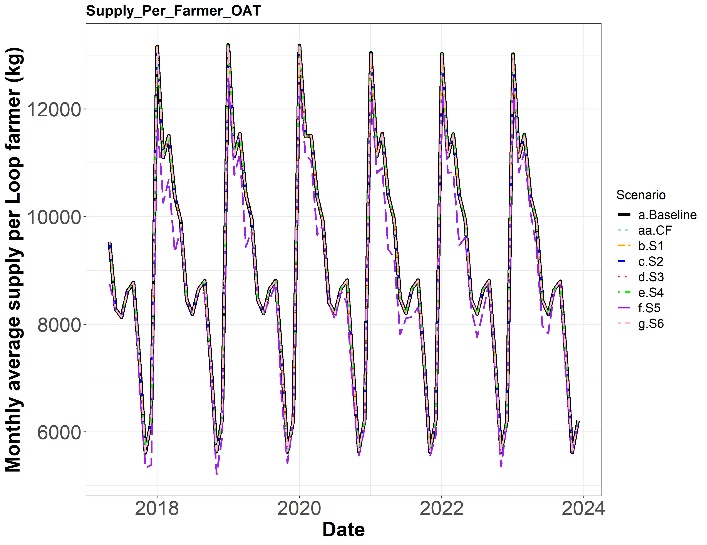

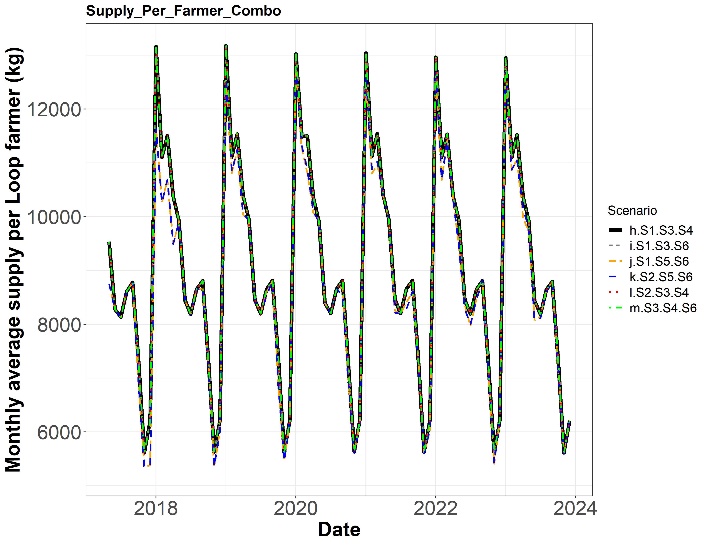


S6 Figure: Monthly average supply per Loop farmers under the once at a time and combo scenarios.


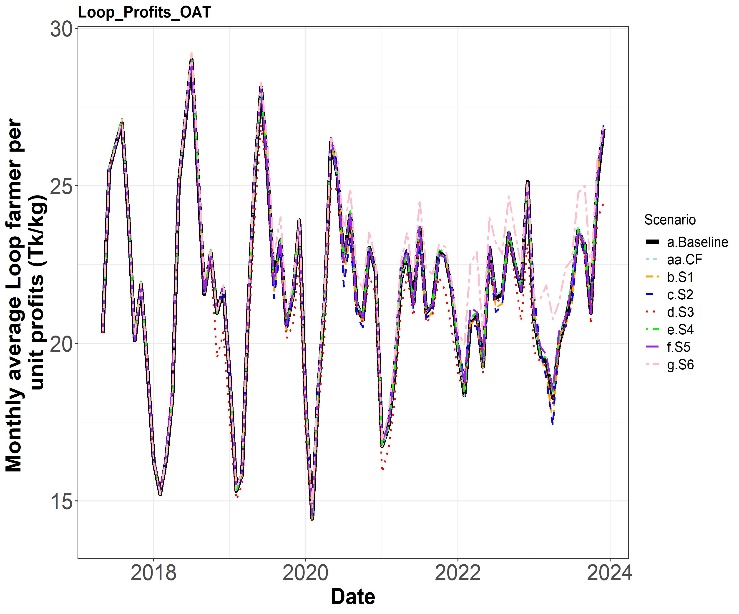

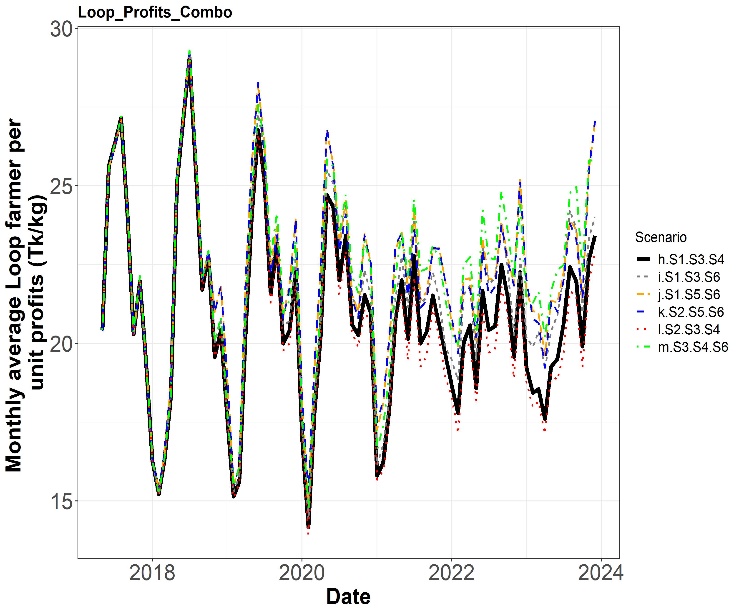


S7 Fig: Monthly average Loop farmer per unit profit (Tk/Kg) farmers under the once at a time and combo scenarios.


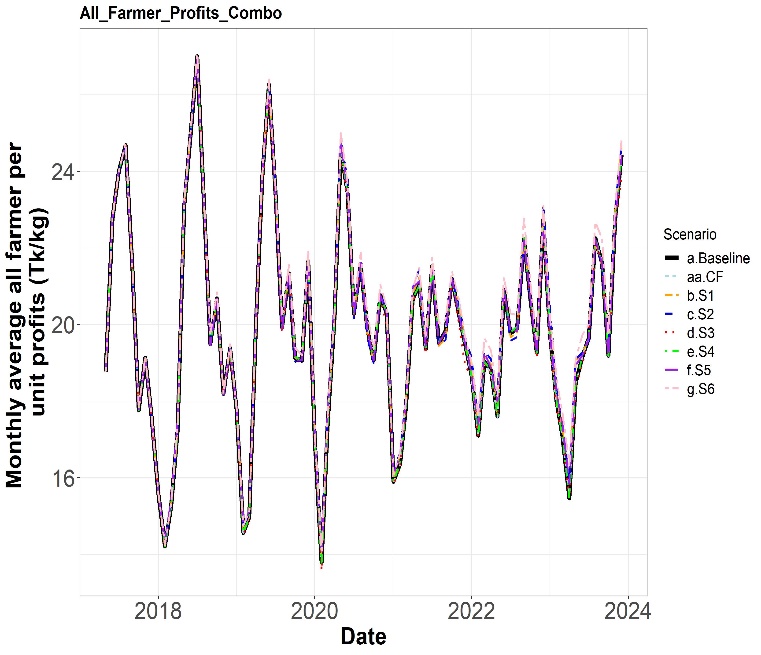

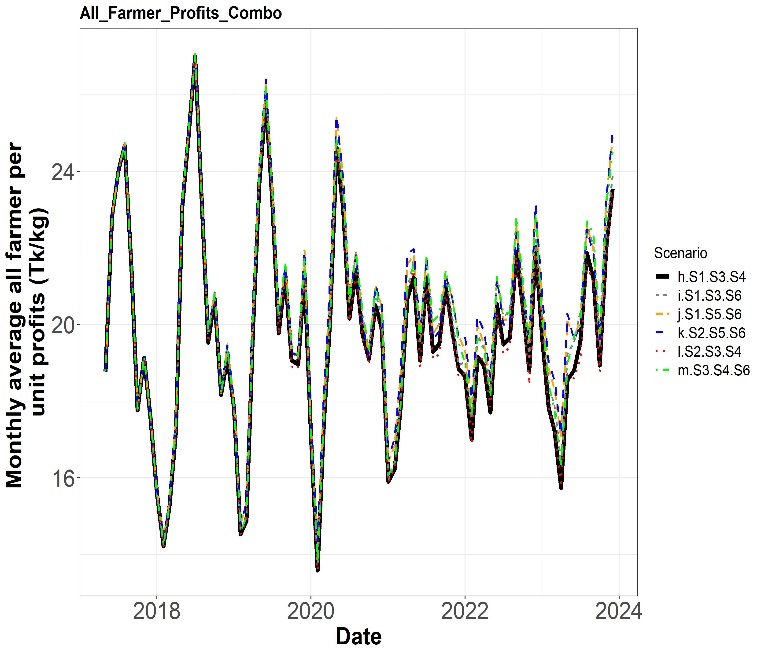


S8 Fig: Monthly average all farmer per unit profits (Tk/Kg) farmers under the once at a time and combo scenarios.


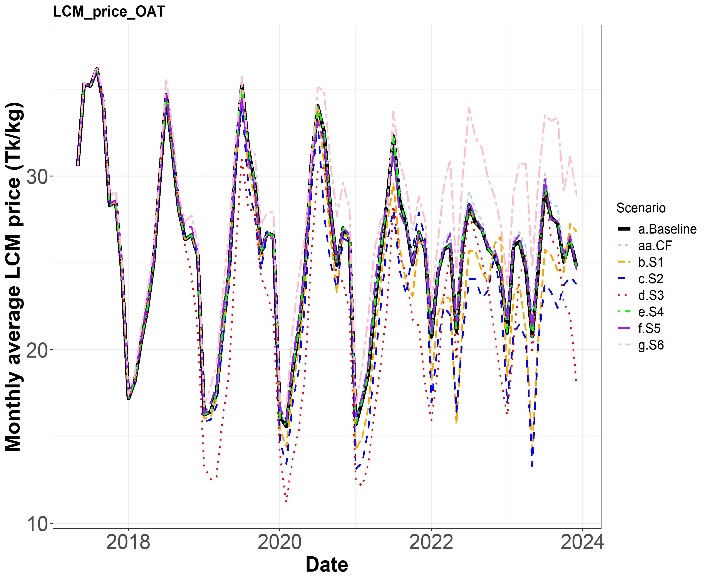

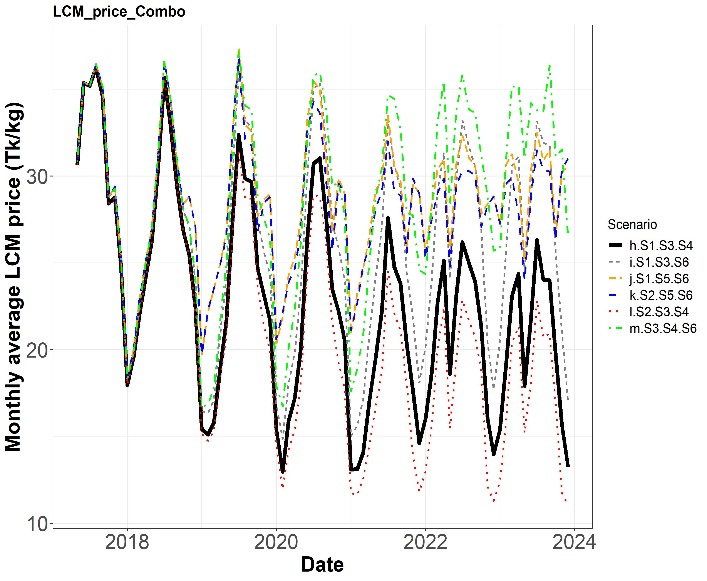


S9 Fig: Monthly average LCM price (Tk/Kg) farmers under the once at a time and combo scenarios.


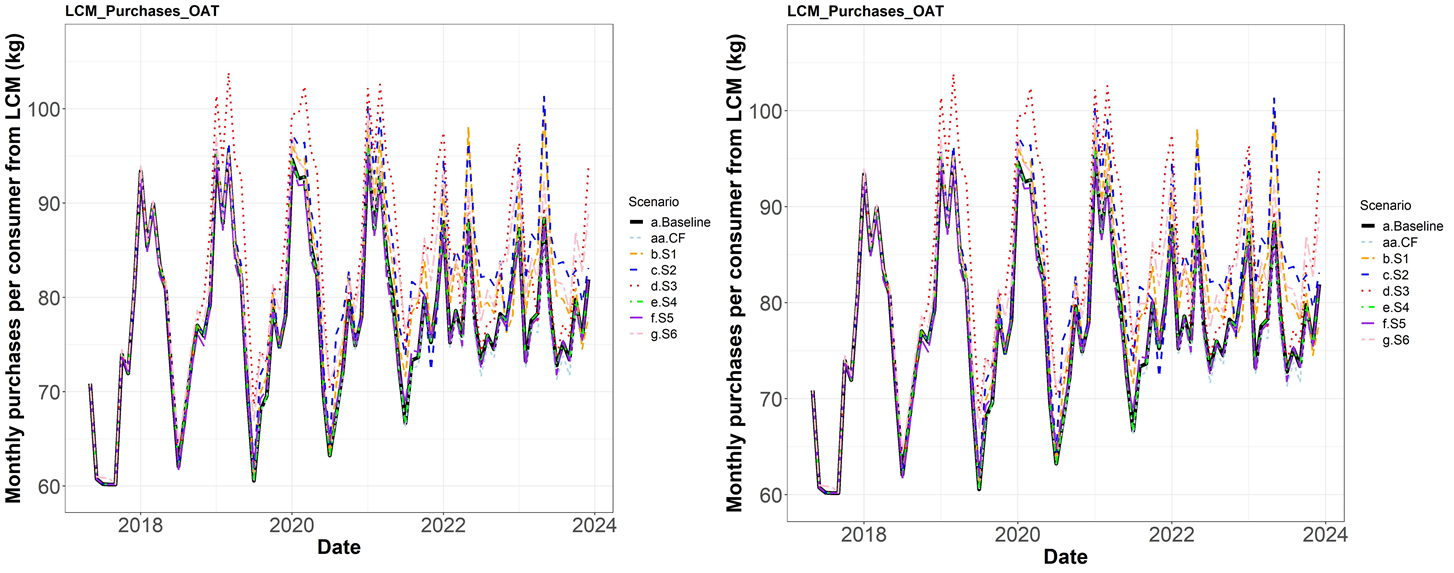


S10 Fig: Monthly average consumer purchases (Kg) from LCM under the once at a time and combo scenarios.
