# Supplementary material for: Identifying value chain trade-offs from fruit and vegetable aggregation services in Bangladesh using a system dynamics approach
Source: PLoS One. 2024 Jan 24;19(1):e0297509. doi: 10.1371/journal.pone.0297509 (PMC10807782; doi:10.1371/journal.pone.0297509)
Supplement: S5 File — (DOCX) [file pone.0297509.s005.docx]

**Supporting information 5: Model equations and parameter values**

"#ofTraders"(t) = "#ofTraders"(t - dt) + (Traders_biflow) * dt {NON-NEGATIVE}

INIT "#ofTraders" = 50

UNITS: person

INFLOWS:

Traders_biflow = IF Profitoftraders>0 AND seasonalLLM>"#ofTraders" THEN 1 ELSE 0

UNITS: People/Days

AggregationforMarket(t) = AggregationforMarket(t - dt) + (LOOPAggregationOutflow - LSMSupplyLOOP - LCMLOOPInFlow - LOOPLLMInFlow) * dt {NON-NEGATIVE}

INIT AggregationforMarket = 0

UNITS: kg

INFLOWS:

LOOPAggregationOutflow = IF ProductionStock*MaxLOOPTrust < DailyTotalAggregationCapacity THEN ProductionStock*MaxLOOPTrust ELSE DailyTotalAggregationCapacity {UNIFLOW}

UNITS: kg/day

OUTFLOWS:

LSMSupplyLOOP = AggregationforMarket*LSMProportionLOOP {UNIFLOW}

UNITS: kg/day

LCMLOOPInFlow = IF LCMProportionLOOP>0 THEN (AggregationforMarket*LCMProportionLOOP) -(AggregationforMarket*LCMProportionLOOP*LCM_wastage) ELSE 0 {UNIFLOW}

UNITS: kg/day

LOOPLLMInFlow = (AggregationforMarket*LLMProportionLOOP) {UNIFLOW}

UNITS: kg/day

ColdstorageVolume(t) = ColdstorageVolume(t - dt) + (VegColdstorageFlow - LOOPColdstorageAggregation - LOOPColdstorageNL - ColdStorageWastage) * dt {NON-NEGATIVE}

INIT ColdstorageVolume = 0

UNITS: kg

INFLOWS:

VegColdstorageFlow = VegForColdstorage {UNIFLOW}

UNITS: kg/day

OUTFLOWS:

LOOPColdstorageAggregation = IF LOOPNetWieghtedProfitPerUnit>ThreshholdProfitforColdstorage THEN MaxLOOPTrust*ColdstorageVolume ELSE 0 {UNIFLOW}

UNITS: kg/day

LOOPColdstorageNL = IF NLNetWieghtedProfitPerUnit>ThreshholdProfitforColdstorage THEN (1-MaxLOOPTrust) *ColdstorageVolume ELSE 0 {UNIFLOW}

UNITS: kg/day

ColdStorageWastage = ColdstorageVolume/ColdstorageShelfLife {UNIFLOW}

UNITS: kg/day

ConsumerGrowthRate(t) = ConsumerGrowthRate(t - dt) + (ConsumerGrowthChange) * dt {NON-NEGATIVE}

INIT ConsumerGrowthRate = 1

UNITS: Dimensionless

INFLOWS:

ConsumerGrowthChange = ConsumerGrowthRate*CGROWTH(ConsumerPercentageGrowthRate)/TotalDays {UNIFLOW}

UNITS: Dimensionless/halfdays

CumLOOPAggregation(t) = CumLOOPAggregation(t - dt) + (Aggregationflow) * dt

INIT CumLOOPAggregation = 0

UNITS: kg

INFLOWS:

Aggregationflow = LOOPAggregationOutflow {UNIFLOW}

UNITS: Kg/day

CumNLFarmersProfitWithoutInvestment(t) = CumNLFarmersProfitWithoutInvestment(t - dt) + (NLProfitChangeWI) * dt

INIT CumNLFarmersProfitWithoutInvestment = 0

UNITS: taka

INFLOWS:

NLProfitChangeWI = NLProfitChangge {UNIFLOW}

UNITS: Taka/Day

CumRetailerCost(t) = CumRetailerCost(t - dt) + (RetailerCostBiflow) * dt

INIT CumRetailerCost = 50

UNITS: taka

INFLOWS:

RetailerCostBiflow = PerRetailerVegExpenditure

UNITS: Taka/Day

CumRetailerRevenue(t) = CumRetailerRevenue(t - dt) + (RetailerRevenueBiflow) * dt

INIT CumRetailerRevenue = 500

UNITS: taka

INFLOWS:

RetailerRevenueBiflow = RevenuePerRetailer

UNITS: Taka/Day

CumulativeLOOPFarmerProfitWithoutInvestment(t) = CumulativeLOOPFarmerProfitWithoutInvestment(t - dt) + (LOOPProfitChangeWI) * dt {NON-NEGATIVE}

INIT CumulativeLOOPFarmerProfitWithoutInvestment = 0

INFLOWS:

LOOPProfitChangeWI = ProfitChangge {UNIFLOW}

CumulativeperLOOP_farmerProfit(t) = CumulativeperLOOP_farmerProfit(t - dt) + (ProfitChangge - Yield_investment) * dt {NON-NEGATIVE}

INIT CumulativeperLOOP_farmerProfit = 0

UNITS: taka

INFLOWS:

ProfitChangge = (LOOPWieghtedProfitPerFarmer/FarmerSupplyFreqLOOP)

UNITS: taka/day

OUTFLOWS:

Yield_investment = IF SeasonChange = 1 AND LoopSeasonalProfitChange > 0 THEN (CumulativeperLOOP_farmerProfit * Yieldinvestment_rate) ELSE 0 {UNIFLOW}

UNITS: Taka/Day

CumulativeperNL_farmerProfit(t) = CumulativeperNL_farmerProfit(t - dt) + (NLProfitChangge - YieldInvestment) * dt

INIT CumulativeperNL_farmerProfit = 0

UNITS: taka

INFLOWS:

NLProfitChangge = (NLWeightedProfitPerFarmer)/FarmerSupplyFreNL

UNITS: taka/day

OUTFLOWS:

YieldInvestment = IF SeasonChangeNL = 1 AND NLSeasonalProfitChange > 0 THEN (CumulativeperNL_farmerProfit * NLYieldinvestmentRate) ELSE 0 {UNIFLOW}

UNITS: Taka/Day

CumulLCMCustomerDemand(t) = CumulLCMCustomerDemand(t - dt) + (CumulLCMDemandFlow) * dt

INIT CumulLCMCustomerDemand = 0.165*4.5

UNITS: kg

INFLOWS:

CumulLCMDemandFlow = LCMPerCustomerDemands*LCMCustomerRoutine {UNIFLOW}

UNITS: Kg/day

CumWeightedAllFarmerProfit(t) = CumWeightedAllFarmerProfit(t - dt) + (AllProfitChange) * dt

INIT CumWeightedAllFarmerProfit = 0

UNITS: taka

INFLOWS:

AllProfitChange = ((LOOPWieghtedProfitPerFarmer*(TrustAndCapacityAdjustedLOOPFarmers/(TrustAndCapacityAdjustedLOOPFarmers+NLFarmersupplyingday)))/FarmerSupplyFreqLOOP)+((NLWeightedProfitPerFarmer*(NLFarmersupplyingday/(TrustAndCapacityAdjustedLOOPFarmers+NLFarmersupplyingday)))/FarmerSupplyFreNL) {UNIFLOW}

UNITS: Taka/Day

LandStockPerFarmer(t) = LandStockPerFarmer(t - dt) {NON-NEGATIVE}

INIT LandStockPerFarmer = 146

UNITS: Decimal

LCMPerCustomerDemands(t) = LCMPerCustomerDemands(t - dt) + (AdjustmentCustomerDemand) * dt

INIT LCMPerCustomerDemands = Reference_Consumer_Demand

UNITS: kg

DOCUMENT: Industry demand adjusts to the indicated demand with a delay, representing the time required for consumers of the good to find substitutes or change their consumption after a change in price. Dimension = Units /Year

INFLOWS:

AdjustmentCustomerDemand = (Indicated_consumer_Demand-LCMPerCustomerDemands)/Demand_adjustment_delay

UNITS: Kg/day

LCMStock(t) = LCMStock(t - dt) + (LCMLOOPInFlow + NLLCMflow - LCMoutflow - LCMWastage) * dt {NON-NEGATIVE}

INIT LCMStock = LCMDailyDemand

UNITS: kg

INFLOWS:

LCMLOOPInFlow = IF LCMProportionLOOP>0 THEN (AggregationforMarket*LCMProportionLOOP) -(AggregationforMarket*LCMProportionLOOP*LCM_wastage) ELSE 0 {UNIFLOW}

UNITS: kg/day

NLLCMflow = IF LCMProportionNL>0 THEN (NLoutflow*LCMProportionNL) -(NLoutflow*LCMProportionNL*NLLCMWastage) ELSE 0 {UNIFLOW}

UNITS: kg/day

OUTFLOWS:

LCMoutflow = LCMDailyDemand {UNIFLOW}

UNITS: kg/day

LCMWastage = LCMStock*LCMWastagerate {UNIFLOW}

UNITS: kg/day

LLMMarketableStock(t) = LLMMarketableStock(t - dt) + (LOOPLLMInFlow + NLLLMinflow - LLMMarketOutFlow - WastageoutflowDT) * dt {NON-NEGATIVE}

INIT LLMMarketableStock = 0

UNITS: kg

INFLOWS:

LOOPLLMInFlow = (AggregationforMarket*LLMProportionLOOP) {UNIFLOW}

UNITS: kg/day

NLLLMinflow = ((NLoutflow*NLProportionLLM)-(NLoutflow*NLProportionLLM*LLMWastage)) {UNIFLOW}

UNITS: kg/day

OUTFLOWS:

LLMMarketOutFlow = LLMDemand {UNIFLOW}

UNITS: Kg/day

WastageoutflowDT = LLMMarketableStock*WastageLLM {UNIFLOW}

UNITS: Kg/day

LLMPref(t) = LLMPref(t - dt) + (Bigmarketprefchange + LLMTrustChange) * dt {NON-NEGATIVE}

INIT LLMPref = 0.75

UNITS: Dimensionless

INFLOWS:

Bigmarketprefchange = IF RevenuePerKGLLM +BestSmalMarketRevenuePerKg = 0 THEN 0 ELSE ((RevenuePerKGLLM/(RevenuePerKGLLM+BestSmalMarketRevenuePerKg))-0.5)/MarketPriceTime

UNITS: Per Day

LLMTrustChange = IF LLMTrust +MaxLocalMarketTrust = 0 THEN 0 ELSE ((LLMTrust/(LLMTrust+MaxLocalMarketTrust))-0.5)/MarketTrustTime

UNITS: Per Day

LongRunPerUnitLOOPProfit(t) = LongRunPerUnitLOOPProfit(t - dt) + (LongRunPerUnitLOOPFlow) * dt

INIT LongRunPerUnitLOOPProfit = 0

INFLOWS:

LongRunPerUnitLOOPFlow = IF TIME <120 THEN 0 ELSE WeightedAvprofitperLOOPfarmerPerUnit

LongRunPerUnitNLProfit(t) = LongRunPerUnitNLProfit(t - dt) + (LongRunPerUnitNLFlow) * dt

INIT LongRunPerUnitNLProfit = 0

INFLOWS:

LongRunPerUnitNLFlow = IF TIME <120 THEN 0 ELSE WeightedAvprofitperNLfarmerPerUnit

LOOPFarmers(t) = LOOPFarmers(t - dt) + (LOOPAdopters) * dt {NON-NEGATIVE}

INIT LOOPFarmers = 33

UNITS: Farmer

INFLOWS:

LOOPAdopters = IF TIME >540 AND CF=1 THEN 0 ELSE (AdoptionWordofMouth+AdoptingExtension*MaxLOOPTrust)

UNITS: Farmer/Days

LOOPPreference(t) = LOOPPreference(t - dt) + (FInancialdrivers) * dt {NON-NEGATIVE}

INIT LOOPPreference = 0.8

INFLOWS:

FInancialdrivers = SMTH1((RandomLOOPprice/(RandomLOOPprice+RandomNLprice)-0.5), PriceTrustTime)

LSMPref(t) = LSMPref(t - dt) + (Smallmarketprechange + LSMTrustChange) * dt {NON-NEGATIVE}

INIT LSMPref = 0.875

UNITS: Dimensionless

INFLOWS:

Smallmarketprechange = IF RevenuePerKGLSM +RevenuePerKGLCM = 0 THEN 0 ELSE ((RevenuePerKGLSM/(RevenuePerKGLCM+RevenuePerKGLSM))-0.5)/MarketPriceTime

UNITS: Per Day

LSMTrustChange = IF LSMIVTrust +LCMTrust = 0 THEN 0 ELSE ((LSMIVTrust/(LSMIVTrust+LCMTrust))-0.5)/MarketTrustTime

UNITS: Per Day

LSMStock(t) = LSMStock(t - dt) + (LSMSupplyLOOP + NLLSMinflow - LSM0utflow - WastageOutflow) * dt {NON-NEGATIVE}

INIT LSMStock = LSMdemand

UNITS: kg

INFLOWS:

LSMSupplyLOOP = AggregationforMarket*LSMProportionLOOP {UNIFLOW}

UNITS: kg/day

NLLSMinflow = IF NLProportionLSM>0 THEN (NLoutflow*NLProportionLSM) -(NLoutflow*NLProportionLSM*LSMWastage) ELSE 0 {UNIFLOW}

UNITS: kg/day

OUTFLOWS:

LSM0utflow = LSMdemand {UNIFLOW}

UNITS: Kg/day

WastageOutflow = LSMStock*(1-LTWastageRate) {UNIFLOW}

UNITS: Kg/day

MarketTrust(t) = MarketTrust(t - dt) + (MarketCapacity + PreferedMarketDistance) * dt

INIT MarketTrust = 0

INFLOWS:

MarketCapacity = 1 {UNIFLOW}

PreferedMarketDistance = 1 {UNIFLOW}

NLMarketableStock(t) = NLMarketableStock(t - dt) + (NLProductionOutflow) * dt {NON-NEGATIVE}

INIT NLMarketableStock = 0

UNITS: kg

INFLOWS:

NLProductionOutflow = NLProductionStock/FarmerSupplyFreNL {UNIFLOW}

UNITS: kg/day

NLProductionStock(t) = NLProductionStock(t - dt) + (NLproductioninflow - NLProductionOutflow - NLGivenawayflow - NLEatenOutflow) * dt {NON-NEGATIVE}

INIT NLProductionStock = NLproductioninflow

UNITS: kg

INFLOWS:

NLproductioninflow = VegLandProportion*NonLOOPFarmers*NLAdjustedYeild*LandStockPerFarmer {UNIFLOW}

UNITS: kg/day

OUTFLOWS:

NLProductionOutflow = NLProductionStock/FarmerSupplyFreNL {UNIFLOW}

UNITS: kg/day

NLGivenawayflow = NLGivenawayPerHH*NLProductionStock {UNIFLOW}

UNITS: kg/day

NLEatenOutflow = NLConsumptionperHH*NLProductionStock {UNIFLOW}

UNITS: kg/day

NonLoopmarketableStock(t) = NonLoopmarketableStock(t - dt) + (NonLoopoutflow + NLMkIntflow - NLoutflow) * dt {NON-NEGATIVE}

INIT NonLoopmarketableStock = 0

UNITS: kg

INFLOWS:

NonLoopoutflow = (ProductionStock*(1-MaxLOOPTrust))+LoopNLCapacityLimitsRunoff {UNIFLOW}

UNITS: kg/day

NLMkIntflow = NLProductionOutflow {UNIFLOW}

UNITS: kg/day

OUTFLOWS:

NLoutflow = NonLoopmarketableStock {UNIFLOW}

UNITS: kg/day

Population(t) = Population (t - dt) + (PopulationIncreasing - PopulationDecrease) * dt {NON-NEGATIVE}

INIT Population = 27000

UNITS: People

INFLOWS:

PopulationIncreasing = BirthRate*Population/(100-DropOut-DeathRate) {UNIFLOW}

UNITS: People/Days

OUTFLOWS:

PopulationDecrease = (Population*DropOut+Population*DeathRate)/100 {UNIFLOW}

UNITS: People/Days

ProductionStock(t) = ProductionStock(t - dt) + (LOOPProductionAvaiableForAggregation - LOOPAggregationOutflow - HHEatenOutFlow - HHGivenAwayOutflow - NonLoopoutflow - VegForColdstorage) * dt {NON-NEGATIVE}

INIT ProductionStock = 1

UNITS: kg

INFLOWS:

LOOPProductionAvaiableForAggregation = VegLandProportionlimit*LandStockPerFarmer*LOOPAdjustedYeild*LOOPFarmers {UNIFLOW}

UNITS: kg/day

OUTFLOWS:

LOOPAggregationOutflow = IF ProductionStock*MaxLOOPTrust < DailyTotalAggregationCapacity THEN ProductionStock*MaxLOOPTrust ELSE DailyTotalAggregationCapacity {UNIFLOW}

UNITS: kg/day

HHEatenOutFlow = ProductionStock*HHEatenperHH {UNIFLOW}

UNITS: kg/day

HHGivenAwayOutflow = GivenwayLOOPPerHH*ProductionStock {UNIFLOW}

UNITS: kg/day

NonLoopoutflow = (ProductionStock*(1-MaxLOOPTrust))+LoopNLCapacityLimitsRunoff {UNIFLOW}

UNITS: kg/day

VegForColdstorage = IF (LOOPNetWieghtedProfitPerUnit>ThreshholdProfitforColdstorage OR ColdstorageSwitch=0) THEN 0 ELSE (IF (0.20*ProductionStock)>ColdStorageRemainingCapacity THEN ColdStorageRemainingCapacity ELSE (0.20*ProductionStock)) {UNIFLOW}

UNITS: kg/day

TotalVeglandstockNL(t) = TotalVeglandstockNL(t - dt) {NON-NEGATIVE}

INIT TotalVeglandstockNL = 66

UNITS: Decimal

VegLandProportion(t) = VegLandProportion(t - dt) + (LandHoldingPerFarmer) * dt {NON-NEGATIVE}

INIT VegLandProportion = 0.6

UNITS: Decimal

INFLOWS:

LandHoldingPerFarmer = (LandHoldingDuringRabi+LandHoldingDuringKharif2+LandHoldingDuringKharif1)

UNITS: Decimal/Days

VegLandProportionNL(t) = VegLandProportionNL(t - dt) + (LandHoldingPerFarmerNL) * dt {NON-NEGATIVE}

INIT VegLandProportionNL = 1

UNITS: Decimal

INFLOWS:

LandHoldingPerFarmerNL = (LandHoldingDuringRabiNL+LandHoldingDuringKharifNL+NLLandHoldingDuringKharif)

UNITS: Decimal/Days

UNITS: People

"#ofLCM" = 3.25

UNITS: Markets

"#OFLLM" = 2

UNITS: Market

"#ofMarket" = 2

UNITS: Markets

UNITS: person

AbsulateDaysPerSeasonKharif_2 = IF Season=2 THEN 122 ELSE 0

AbsulateDaysPerSeasonKharif1 = IF Season = 1 THEN 92 ELSE 0

AbsulateDaysPerSeasonRabi = IF Season =3 THEN 155 ELSE 0

ActualLCMOutFlowPerConsumer = LCMoutflow/(LCMRetailar*CustomersPerRetailer)

UNITS: kg

UNITS: taka

AdjustedRetaileLCM = RetailerLCM*EffectofExpected_ProfitDesiredCap

UNITS: People

AdoptingExtension = NonLOOPFarmers*ExtensionSwitch

UNITS: Dimensionless

AdoptionWordofMouth = (ContactRate*WordOfMouthEffectiveness*NonLOOPFarmers*LOOPFarmers*MaxLOOPTrust)/Population

UNITS: Dimensionless

AggregationFee = IF TIME <=150 THEN 1 ELSE 0.50

UNITS: taka

AggregationFeePerfarmer = AggregationFee*PerFarmerSupplyingLSM

UNITS: taka

AggregatorCom% = IF TIME <=309 THEN 0.50 ELSE (IF (TIME >309 AND TIME <=540) THEN 0.25 ELSE 0.50)

UNITS: taka

Aggregators_capacity = 2000

UNITS: kg

AlamshadhuCapacity = RANDOM(1200, 2000, 13)

UNITS: kg

AlamshadhuDriverCost = RANDOM(200, 300, 12)

UNITS: taka

Alamshadhulifetime = 7

UNITS: Years

AlamshadhuPrice = 150000

UNITS: taka

AllProfit = NLWeightedProfitPerFarmer+LOOPWieghtedProfitPerFarmer

UNITS: taka

AverageHHMember = 4.5

UNITS: persons

Base = 0

UNITS: Dimensionless

BaseFuelCost = 66

UNITS: km/litre

BaseFuelprice = 65

UNITS: taka

BaseProfit = 1.5

UNITS: Taka/Kg

BaseYield = ((RabiYeildPerSeason*DaysPerSeasonRabi)+(Kharif1YieldPerSeason*DaysPerSeasonKharif1)+(Kharif2YeildPerSeason*DaysPerSeasonKharif2))

UNITS: Kg/Decimal

BestSmalMarketRevenuePerKg = MAX(RevenuePerKGLSM, RevenuePerKGLCM)

UNITS: taka

BirthRate = 1.8/DaysPerYear

UNITS: person

BuyingCapacity = 2000

UNITS: kg

CF = 0

ColdstorageatCapacity = IF ColdstorageVolume>=ColdStorageCapacity THEN 1 ELSE 0

UNITS: kg

ColdStorageCapacity = 300000

UNITS: kg

ColdStorageRemainingCapacity = ColdStorageCapacity-ColdstorageVolume

UNITS: kg

ColdstorageShelfLife = 21

UNITS: Days

ColdstorageSwitch = 0

UNITS: Dimensionless

ConsumerPercentageGrowthRate = 2.5

UNITS: Dimensionless

UNITS: Taka/Kg

ContactRate = 2

UNITS: Dimensionless

Costofproduce = 15

UNITS: taka/kg

CostPerDayPerAggregator = CostPerTripAlamshadhu*Vehicle#PerDay

UNITS: taka

CostPerRV = RANDOM(100, 400, 251)

UNITS: taka

CostPerTripAlamshadhu = FuelCostPerTrip_A+MaintanenceCostPerTrip_A+PerTripDepriciationCost_A+AlamshadhuDriverCost+PerTripProfit

UNITS: taka

CoveragePerceptionTime = 7

UNITS: days

CoverageperceptiontimeLLCM = 7

CoverageperceptiontimeLLSM = 7

CustomersPerRetailer = InitialRetailCus_tomersPerRetailer*ConsumerGrowthRate

UNITS: People/day

DailyAggregationComperfarmer = LOOPPerFarmerVolume*AggregatorCom%

UNITS: taka

DailyConsumptionPerCustomer = (CumulLCMCustomerDemand/TIME/AverageHHMember)*1000

UNITS: Grams

DailyDepreciation = YearlyDepriciationCost/365

DailyDepreciation_A = YearlyDepriciationCost_A/365

UNITS: taka

DailyLabourCost = 500

UNITS: taka

dailyloancost = loaninstallment/7

UNITS: taka

DailyMaintainenceCost = MonthlyMaintainenceCost/30

DailyMaintainenceCost_A = MonthlyMaintainenceCost_A/30

UNITS: taka

DailyMarketFeePerFarmerLOOP = ((YearlyMarketwastage*ExpectedPriceLocal)/365)

UNITS: taka

DailyMarketWastages = LOOPDholta+LOOPTola

UNITS: Dimensionless

DailyPerFarmerTransportCost = FarmerPerDayTransportCost*(1-TransportSubsidyAll%)

UNITS: taka

DailyTotalAggregationCapacity = IF TIME<300 THEN Aggregators_capacity*NumberOfAggregators ELSE (Aggregators_capacity*NumberOfAggregators)/2

UNITS: kg

DailyWeightedAllFarmersProfit = (LOOPWieghtedProfitPerFarmer*(TrustAndCapacityAdjustedLOOPFarmers/(TrustAndCapacityAdjustedLOOPFarmers+NLFarmersupplyingday)))+(NLWeightedProfitPerFarmer*(NLFarmersupplyingday/(TrustAndCapacityAdjustedLOOPFarmers+NLFarmersupplyingday)))

UNITS: taka

DaysPerSeasonKharif1 = IF Season=1 THEN 1/92 ELSE 0

UNITS: Days

DaysPerSeasonKharif2 = IF Season=2 THEN 1/122 ELSE 0

UNITS: Days

DaysPerSeasonRabi = IF Season=3 THEN 1/151 ELSE 0

UNITS: Days

DaysPerYear = 365

UNITS: Days

Daytransportcost = TransportcostPerTripTruck/Tripdays

UNITS: taka

DeathRate = 0.54/DaysPerYear

UNITS: person

DefaultLOOPTrust = 0.8

UNITS: Dimensionless

DefaultMinLOOPLLMPref = 0.80

UNITS: Dimensionless

Demand_adjustment_delay = 3

UNITS: Days

DOCUMENT: The average time required for consumer demand to respond to a change in price. Units = Years

Demand_Curve_Slope = (-Reference_Consumer_Demand*Reference_consumer_Demand_Elasticity)/Reference_Price

UNITS: Dimensionless

DOCUMENT: The slope of the industry demand curve, as a function of the price elasticity at the reference price level Units = Unit*Units/($*Year)

DholtaLLM = 0.05

UNITS: Percent

DholtaLSM = 0.06

UNITS: Percent

DiegelCost = 7

UNITS: km/litre

Distancecovered = RANDOM(5, 30, 14)

UNITS: km

DriverCost = RANDOM(4000, 5000, 103)

UNITS: taka

DropOut = 0.01/DaysPerYear

UNITS: person

DTWastageenroute = RANDOM(0.05, 0.1, 253)

UNITS: Dimensionless

EffectiveOfInvent_oryCoverageOnPrice = PerceivedInventoryCoverage^SensivityOfPriceto_InventoryCoverage

UNITS: Dimensionless

EffectiveOfInvent_oryCoverageOnPriceLCM = PerceiveinventorycoverageLCM^SensivityOfPriceto_InventoryCoverageLCM

UNITS: Dimensionless

EffectiveOfInvent_oryCoverageOnPriceLSM = PerceiveinventorycoverageLSM^SensivityOfPriceto_InventoryCoverageLSM

UNITS: Dimensionless

EffectofCotsOnPrice = 1+SensivityOfPriceCots*((LLMBuyingCost/TradersExpectedPrice)-1)

UNITS: Dimensionless

EffectofExpected_ProfitDesiredCap = GRAPH(SMTH1(InvestmentExpectedProfitability,ProfitPerceptionTime))

(-1.000, 0.112), (-0.500, 0.4045), (0.000, 1.172), (0.500, 1.479), (1.000, 1.600)

UNITS: Dimensionless

EffectofExpectedProfitOnLOOPTrust = GRAPH(LOOPExpectedProfitabilty)

(-1.000, 0.000), (-0.500, 0.300), (0.000, 1.000), (0.500, 1.500), (1.000, 1.600)

UNITS: Dimensionless

EffectofExpectedProfitOnMinLOOPLLM = GRAPH(SmallMarketLongRunExpectedProfitability)

(-1.000, 1.000), (-0.500, 1.000), (0.000, 1.000), (0.500, 1.500), (1.000, 1.600)

UNITS: Dimensionless

EffecttocostsonpriceLCM = 1+SensivityOfPriceCostLCM*((LCMbuyingcost/LCMexpectedprice)-1)

UNITS: Dimensionless

EffecttocostsonpriceLSM = 1+SensivityOfPriceCostLSM*((LSMbuyingcost/LSMexpectedprice)-1)

UNITS: Dimensionless

ExpectedLongRunCost = CumRetailerCost-DELAY(CumRetailerCost, 1)

UNITS: taka

ExpectedLongRunRevenue = CumRetailerRevenue-DELAY(CumRetailerRevenue, 1)

UNITS: taka

Ext1 = 1

UNITS: Dimensionless

Ext2 = 0

UNITS: Dimensionless

ExtEffectivenessValue = (IF Base=1 THEN 0 ELSE (IF Ext1=1 THEN 1 ELSE (IF Ext2=1 THEN 2 ELSE 0)))

UNITS: Dimensionless

ExtensionEffectiveness = IF TIME >309 AND TIME<540 THEN 0.00054 ELSE 0.00030

UNITS: Dimensionless

ExtensionSwitch = IF TIME <=540 THEN ExtensionEffectiveness ELSE ExtensionEffectiveness*ExtEffectivenessValue

UNITS: Farmer

FarmerpercievedIVLCM = SMTH1(InventoryCoverageLCM, FarmerSupplyFreqLOOP)

UNITS: Dimensionless

FarmerpercievedIVLLM = SMTH1(InventoryCoverageLLM, FarmerSupplyFreqLOOP)

UNITS: Dimensionless

farmerpercievedIVLSM = SMTH1(InventoryCoverageLSM, FarmerSupplyFreqLOOP)

UNITS: Dimensionless

FarmerPerDayTransportCost = PerKGTAlamshadhuCostLCM*LOOPPerFarmerVolume

UNITS: taka

FarmerRevenueLLM = TradersBuyingPriceLLM*LLMMarketOutFlow

UNITS: taka

FarmerSuppllyingLSM = IF (TrustAndCapacityAdjustedLOOPFarmers*LSMProportionLOOP)<1 THEN 1 ELSE (TrustAndCapacityAdjustedLOOPFarmers*LSMProportionLOOP)

UNITS: Farmer

FarmerSupplyFreNL = (RabiSupplyFreq+Kharif1SupplyFreq+Kharif2SupplyFreq)

UNITS: Dimensionless

FarmerSupplyFreqLOOP = IF TIME >308 THEN (RabiSupplyFreq+Kharif1SupplyFreq+Kharif2SupplyFreq) ELSE (RabiSupplyFreq+Kharif1SupplyFreq+Kharif2SupplyFreq)

UNITS: Dimensionless

FerryCost = RANDOM(600, 1000, 101)

UNITS: taka

FuelCostPerTrip = PerTripFuelConsumption*BaseFuelCost

FuelCostPerTrip_A = PerTripConsumption*BaseFuelprice

FuelEconomy = 4

UNITS: km/litre

GivenwayLOOPPerHH = 0.06

UNITS: Percent

HHEatenperHH = 0.07

UNITS: Percent

Indicated_consumer_Demand = MIN(Maximum_Consumption,Reference_Consumer_Demand)*MAX(0,1+Demand_Curve_Slope*(RetailerSellingPrice-Reference_Price)/Reference_Consumer_Demand)

UNITS: Kilograms

DOCUMENT: The indicated demand for the commodity given the current price. Indicated demand is the demand consumers would like given the current price. Actual demand adjusts to indicated demand with a delay. The demand curve is linear, with slope set so that the elasticity of demand at the reference price is equal to the reference industry demand elasticity, set by the user. Units = Units / Year

InitialRetailCus_tomersPerRetailer = RANDOM(100, 120, 251)

UNITS: People/day

InputSubsidy% = IF InputSwith=0 THEN 0 ELSE 0.5

InputSwith = 0

UNITS: Dimensionless

InventoryCoverageLCM = IF LCMoutflow =0 THEN 1 ELSE LCMStock/LCMoutflow

UNITS: Dimensionless

InventoryCoverageLLM = IF LLMMarketOutFlow=0 THEN 1 ELSE LLMMarketableStock/LLMMarketOutFlow

UNITS: Dimensionless

InventoryCoverageLSM = IF LSM0utflow=0 THEN 1 ELSE LSMStock/LSM0utflow

UNITS: Dimensionless

InvestmentExpectedProfitability = IF ExpectedLongRunCost = 0 OR ExpectedLongRunRevenue =0 THEN 1 ELSE (ExpectedLongRunRevenue-ExpectedLongRunCost)/ExpectedLongRunCost

UNITS: taka

InvestmentproportionCost = LOOPYeildInvestment/LOOPtotaldaycost

UNITS: taka

InvestmentproportionCostNL = NLYeildInvestment/TotalNLdaycost

UNITS: taka

Kharif1SupplyFreq = IF Season = 1 THEN 5 ELSE 0

UNITS: days

Kharif1YieldPerSeason = IF Season = 1 THEN 80 ELSE 0

UNITS: Kg/Decimal

Kharif2SupplyFreq = IF Season = 2 THEN 5 ELSE 0

UNITS: days

Kharif2YeildPerSeason = IF Season=2 THEN 90 ELSE 0

UNITS: Kg/Decimal

Laborincrease = 2

LabourCapacity = 500

UNITS: kg/person

LabourHourlyWage = 400/5

UNITS: taka/hour

LabourRate = 500

UNITS: taka

LabourRequired = BuyingCapacity/LabourCapacity

UNITS: kg/person

LandHoldingDuringKharif1 = IF Season=1 THEN -0.0015 ELSE 0

UNITS: Decimal

LandHoldingDuringKharif2 = IF Season=2 THEN 0.001 ELSE 0

UNITS: Decimal

LandHoldingDuringKharifNL = IF Season=1 THEN -0.003 ELSE 0

UNITS: Decimal

LandHoldingDuringRabi = IF Season=3 THEN 0.0016 ELSE 0

UNITS: Decimal

LandHoldingDuringRabiNL = IF Season=3 THEN 0.001 ELSE 0

UNITS: Decimal

LCM_wastage = 0.05

UNITS: kg

LCMbuyingcost = (MarketcostLCM+TranportCostPerUnitPerFarmer+PerKgLaborcostLCM+VegetablesCostLCM+WastagecostLCM)

UNITS: taka/kg

LCMCustomerRoutine = 2/7

UNITS: Days

LCMDailyDemand = LCMPerRetailerDemands*LCMRetailar

UNITS: kg

LCMexpectedprice = ActualSalePriceLCM

UNITS: Dimensionless

LCMfarmerrevenue = LCMoutflow*TraderbuyingpriceLCM

UNITS: taka

LCMLOOPfarmerProfit = IF LCMLOOPperfarmerRevday=0 THEN 0 ELSE LCMLOOPperfarmerRevday-(DailyAggregationComperfarmer+DailyMarketFeePerFarmerLOOP+(DailyPerFarmerTransportCost*(1-TransportSubsidyLCM%)))

UNITS: taka

LCMLOOPFarmerProfitPerUnit = IF LOOPLCMOutflow=0 OR LCMLOOPfarmerProfit=0 THEN 0 ELSE (LCMLOOPfarmerProfit*LCMProportionLOOP*TrustAndCapacityAdjustedLOOPFarmers)/LOOPLCMOutflow

UNITS: Taka/Kg/farmer

LCMLOOPperfarmerRevday = IF (TrustAndCapacityAdjustedLOOPFarmers*LCMProportionLOOP)=0 THEN 0 ELSE LOOPfarmerRevLCM/(TrustAndCapacityAdjustedLOOPFarmers*LCMProportionLOOP)

UNITS: taka

LCMMargin = LCMRetailerSalesCost+LCMRetailerNetProfitPerKG

UNITS: taka/Kg

LCMNLPerfarmerProfitPerUnit = IF NLLCMOutFlow=0 THEN 0 ELSE (NLfarmerProfitLCM*LCMProportionNL*NLFarmersupplyingday)/NLLCMOutFlow

UNITS: Taka/Kg/Farmer

LCMPerRetailerDemands = (LCMPerCustomerDemands*CustomersPerRetailer)

UNITS: Kilograms

LCMProportion = IF TotalMarketInflow=0 THEN 0 ELSE (TotalLCMflow*100)/TotalMarketInflow

UNITS: Dimensionless

LCMProportionLOOP = IF LCMSupplyScenario=1 THEN 0.30 ELSE (1-LSMPrefUse)*ProportionToRemainingMarket

UNITS: Dimensionless

LCMProportionNL = 0.10

UNITS: Percent

LCMRetailar = AdjustedRetaileLCM*"#ofLCM"

UNITS: People

LCMRetailerFee = 1

UNITS: taka

LCMRetailerLaborCost = 1

UNITS: Taka/Kg

LCMRetailerNetProfitPerKG = BaseProfit+(LCMPerCustomerDemands/Reference_Consumer_Demand)

UNITS: Taka/Kg

LCMRetailerSalesCost = LCMRetailerFee+LCMRetailerLaborCost+RetailerTranspostCost

UNITS: Taka/Kg

LCMRevenue = LCMexpectedprice*LCMoutflow

UNITS: taka

LCMSupplyScenario = IF TIME<=540 THEN 0 ELSE SupplySwitch

UNITS: Dimensionless

LCMTrust = GRAPH(FarmerpercievedIVLCM)

(0.000, 0.981), (0.100, 0.963), (0.200, 0.907), (0.300, 0.851), (0.400, 0.800), (0.500, 0.726), (0.600, 0.656), (0.700, 0.586), (0.800, 0.488), (0.900, 0.363), (1.000, 0.000)

UNITS: Dimensionless

LCMWastagerate = 0.05

UNITS: Dimensionless

LFertilizersubsidyratio = 0.5

UNITS: Dimensionless

LLMBuyingCost = (MarketCostLLM+PerKgLaborCostLLM+VegetablesPerKgCostLLM+WastageCostLLM+TranportCostPerUnitPerFarmer)

UNITS: Taka/Kg

LLMCapacity = 4000

UNITS: kg

LLMDemand = LLMCapacity*"#OFLLM"*"#LLMTraders"

UNITS: Kg

LLMLOOPperfarmerProfit = LLMPerLOOPfarmerRevperday-(DailyAggregationComperfarmer+DailyMarketFeePerFarmerLOOP+DailyPerFarmerTransportCost)

UNITS: taka

LLMNLOutFlow = LLMMarketOutFlow-LOOPLLMOutflow

UNITS: kg

LLMNLProfitPerUnit = IF LLMNLOutFlow=0 THEN 0 ELSE (LLMPerFarmerProfitNL*NLProportionLLM*NLFarmersupplyingday)/LLMNLOutFlow

UNITS: Taka/Kg/Farmer

LLMOutflowProLOOP = IF (LOOPLLMInFlow+NLLLMinflow)=0 THEN 0 ELSE LOOPLLMInFlow/(LOOPLLMInFlow+NLLLMinflow)

UNITS: Dimensionless

LLMPerFarmerProfitNL = NLPerFarmerRev-(NLDailyMarketFeePerFarmer+NLDailyTransportCost)

UNITS: taka

LLMPerLOOPfarmerRevperday = IF (TrustAndCapacityAdjustedLOOPFarmers*LLMProportionLOOP)=0 THEN 0 ELSE (LOOPFarmerRevenue/(TrustAndCapacityAdjustedLOOPFarmers*LLMProportionLOOP))

UNITS: taka

LLMProportion = IF TotalMarketInflow=0 THEN 0 ELSE (TotalLLMinflow*100)/TotalMarketInflow

UNITS: Dimensionless

LLMProportionLOOP = IF LLMSupplyscenario >1 THEN 1 ELSE LLMSupplyscenario

UNITS: Dimensionless

LLMProportionPrototype = IF LLMPref<MinLOOPLLMPref THEN MinLOOPLLMPref ELSE LLMPref

UNITS: Dimensionless

LLMrevenue = TradersExpectedPrice*LLMMarketOutFlow*(1-DTWastageenroute)

UNITS: taka

LLMSupplyscenario = IF LCMSupplyScenario=1 THEN 0.70 ELSE LLMProportionPrototype

UNITS: Dimensionless

LLMTrust = GRAPH(FarmerpercievedIVLLM)

(0.000, 1.000), (0.100, 0.930), (0.200, 0.912), (0.300, 0.888), (0.400, 0.870), (0.500, 0.837), (0.600, 0.781), (0.700, 0.726), (0.800, 0.600), (0.900, 0.451), (1.000, 0.000)

UNITS: Dimensionless

LLMWastage = 0.05

UNITS: Percent

loaninstallment = 1142

UNITS: taka/week

LongRunProfit = SMTH1(ProfitPerretailer - DELAY(ProfitPerretailer, ProfitPerceptionTime), ProfitPerceptionTime)

UNITS: taka

LongRunRevPerKgLLM = SMTH1(RevenuePerKGLLM, 25)

UNITS: Taka/Kg

LongRunRevPerKgSmallMarket = SMTH1(BestSmalMarketRevenuePerKg, 25)

UNITS: Taka/Kg

LOOOthers = LOGNORMAL(15, 37, 32)*LabourHourlyWage

UNITS: taka

LOOP%SoldLLM = IF TotalLOOPSales=0 THEN 0 ELSE LOOPLLMOutflow/TotalLOOPSales

UNITS: Dimensionless

LOOPAdjustedYeild = (BaseYield*LOOPYieldImprovement)+BaseYield

UNITS: kg

LOOPAggregationCost = LOOPWaitingcost+LOOPAggregationFee+LOOPTransportCost

UNITS: taka

LOOPAggregationFee = 0.50

UNITS: taka/kg

LoopCapacityLimitedFarmersProp = IF MaxLOOPTrust*ProductionStock = 0 THEN 0 ELSE LoopNLCapacityLimitsRunoff/(MaxLOOPTrust*ProductionStock)

UNITS: Dimensionless

LOOPCostReductionRate = 1

UNITS: Percent

LOOPDailyAggrega_tionCostPerFarmer = (DailyAggregationComperfarmer+LOOPDailyTransportcostPerfarmers)

UNITS: taka

LOOPDailyTransportCostPerFarmerPerUnit = IF LOOPLLMOutflow=0 OR LOOPDailyTransportcostPerfarmers=0 THEN 0 ELSE (LOOPDailyTransportcostPerfarmers*LLMProportionLOOP*TrustAndCapacityAdjustedLOOPFarmers)/LOOPLLMOutflow

UNITS: taka

LOOPDailyTransportcostPerfarmers = IF TIME <=540 THEN DailyPerFarmerTransportCost ELSE DailyPerFarmerTransportCost

UNITS: taka

LOOPDholta = .05

UNITS: Dimensionless

LOOPDropOut = LOOPFarmers*LOOPDropOutRate

UNITS: People

LOOPDropOutRate = 1.1/DaysPerYear

LOOPExpectedProfitabilty = IF LongRunPerUnitLOOPProfit=0 THEN 0 ELSE (LongRunPerUnitLOOPProfit-LongRunPerUnitNLProfit)/LongRunPerUnitLOOPProfit

UNITS: Dimensionless

LOOPFarmerRevenue = FarmerRevenueLLM*ProportionLOOPLLMSS

UNITS: taka

LOOPfarmerRevLCM = LCMfarmerrevenue*ProportionLOOPSSLCM

UNITS: taka

LOOPFarmersSupplyingDay = LOOPFarmers/FarmerSupplyFreqLOOP

UNITS: People

LOOPFertilizer = (LOGNORMAL(11883, 8245, 10)/YearToDayConverter)

UNITS: taka/day

LOOPHarvesting = LOGNORMAL(17.56, 16.9, 31)*LabourHourlyWage

UNITS: taka

LOOPHilling = LOGNORMAL(4.39, 5.15, 27)*LabourHourlyWage

UNITS: taka

LOOPHormone = LOGNORMAL(1114, 1003, 14)/YearToDayConverter

UNITS: taka/day

LOOPIrrigation = LOGNORMAL(2708, 1713, 12)/YearToDayConverter

UNITS: taka/day

LOOPKharif1Weighting = 0.00001

UNITS: Dimensionless

LOOPLCMOutflow = IF (NLLCMflow+LCMLOOPInFlow)=0 THEN 0 ELSE LCMoutflow*(LCMLOOPInFlow/(NLLCMflow+LCMLOOPInFlow))

UNITS: kg

LOOPLLMOutflow = LLMMarketOutFlow*LLMOutflowProLOOP

UNITS: kg

LOOPLLMPerFarmerProfitPerUnit = IF LOOPLLMOutflow=0 OR LLMLOOPperfarmerProfit=0 THEN 0 ELSE ((LLMLOOPperfarmerProfit*LLMProportionLOOP*TrustAndCapacityAdjustedLOOPFarmers)/LOOPLLMOutflow)

UNITS: Taka/Kg/Farmer

LOOPLSMOutflow = IF (NLLSMinflow+LSMSupplyLOOP)=0 THEN 0 ELSE LSM0utflow*(LSMSupplyLOOP/(NLLSMinflow+LSMSupplyLOOP))

UNITS: kg

LOOPManure = LOGNORMAL(2167, 1680, 13)/YearToDayConverter

UNITS: taka/day

LOOPNetWieghtedProfitPerUnit = (WeightedAvprofitperLOOPfarmerPerUnit-PerUnitInputCostLOOP)

UNITS: Taka/Kg

LoopNLCapacityLimitsRunoff = IF MaxLOOPTrust*ProductionStock < DailyTotalAggregationCapacity THEN 0 ELSE (MaxLOOPTrust*ProductionStock) - DailyTotalAggregationCapacity

LOOPNLSupplyingPerDay = LOOPFarmersSupplyingDay-TrustAndCapacityAdjustedLOOPFarmers

UNITS: People

LOOPperdaycost = (SMTH1(LOOPSeeds+LOOPFertilizer+LOOPPesticides+LOOPIrrigation+LOOPManure+LOOPTractor+LOOPRota+LOOPHormone, 30))*(1-InputSubsidy%)

UNITS: taka

LOOPperdaylaborcost = (LOOPPloughing+LOOPPlanting+LOOPHilling+LOOPWeeding+LOOPWatering+LOOPHarvesting+LOOOthers+LOOPpest)/YearToDayConverter

UNITS: taka

LOOPPerFarmerVolume = LOOPAggregationOutflow/(TrustAndCapacityAdjustedLOOPFarmers)

UNITS: kg

LOOPpest = LOGNORMAL(.78, 2.85, 30)*LabourHourlyWage

UNITS: taka

LOOPPesticides = LOGNORMAL(5254, 4615, 11)/YearToDayConverter

UNITS: taka/day

LOOPPlanting = LOGNORMAL(4.14, 4.24, 26)*LabourHourlyWage

UNITS: taka

LOOPPloughing = LOGNORMAL(3.87, 3.83, 25)*LabourHourlyWage

UNITS: taka

LOOPProportion = (TotalLOOPSales*100)/TotalSales

UNITS: kg

LOOPProportionDTOutflow = IF TotalLLMinflow=0 THEN 0 ELSE LOOPLLMInFlow/TotalLLMinflow

UNITS: kg

LOOPProportionLLM = 0.83

UNITS: Percent

LOOPPropostion = IF TotalLOOPMarketInflow=0 THEN 0 ELSE (TotalLOOPMarketInflow*100)/TotalMarketInflow

UNITS: kg

LOOPRabiKharif2Weighting = 0.00001

UNITS: Dimensionless

LOOPReductionCost = LOOPtotaldaycost-(LOOPtotaldaycost*LOOPReductionRate)

LOOPReductionRate = 0.40

UNITS: %

LOOPRevenueLSM = ProportionLOOPSSLSM*LSMRevenue

UNITS: taka

LOOPRota = LOGNORMAL(444, 545, 16)/YearToDayConverter

UNITS: taka/day

LoopSalesPerFarmer = TotalLOOPSales/TrustAndCapacityAdjustedLOOPFarmers

UNITS: kg

LoopSeasonalProfitChange = CumulativeperLOOP_farmerProfit-DELAY(CumulativeperLOOP_farmerProfit, NumberOfDaysPerSeason)

LOOPSeeds = LOGNORMAL(4001, 3815, 9)/YearToDayConverter

UNITS: taka/day

LOOPTola = .04

UNITS: Dimensionless

LOOPtotaldaycost = LOOPperdaycost+LOOPperdaylaborcost

UNITS: taka

LOOPTractor = LOGNORMAL(1012, 846, 15)/YearToDayConverter

UNITS: taka/day

LOOPTransportCost = 0.83

UNITS: taka/kg

LOOPWaitingcost = LOOPwaitingtime*DailyLabourCost/Volumeperfarmer

UNITS: taka

LOOPwaitingtime = RANDOM(5,60, 256)/60/24

UNITS: Days

LOOPWatering = LOGNORMAL(.85, 2.61, 29)*LabourHourlyWage

UNITS: taka

LOOPWeeding = LOGNORMAL(16.22, 15.6, 28)*LabourHourlyWage

UNITS: taka

LOOPWieghtedDailyProfitPerFarmer = LOOPWieghtedProfitPerFarmer

UNITS: taka

LOOPWieghtedProfitPerFarmer = IF TotalLOOPSales=0 THEN 0 ELSE ((LLMLOOPperfarmerProfit*(LOOPLLMOutflow/TotalLOOPSales))+(LSMLOOPfarmerProfit*(LOOPLSMOutflow/TotalLOOPSales))+(LCMLOOPfarmerProfit*(LOOPLCMOutflow/TotalLOOPSales)))-LOOPtotaldaycost

UNITS: Taka

LOOPYearlyTransportCost = CostPerTripAlamshadhu*YearlyMarketSupplyFre

UNITS: taka

LOOPYeildInvestment = Yield_investment/NumberOfDaysPerSeason

UNITS: taka

LOOPYieldImprovement = GRAPH(InvestmentproportionCost)

LSMbuyingcost = (MarketcostLSM+TranportCostPerUnitPerFarmer+PerKgLaborcostLSM+VegetablesCostLSM+WastagecostLSM)

UNITS: taka/kg

LSMdemand = LSMTcapacity*TotalLSMtraders

UNITS: kg

LSMexpectedprice = ActualsalesPriceLSM

UNITS: Dimensionless

LSMIVTrust = GRAPH(farmerpercievedIVLSM)

(0.000, 0.995), (0.100, 0.9513), (0.200, 0.917111111111), (0.300, 0.886133333333), (0.400, 0.831933333333), (0.500, 0.754), (0.600, 0.677), (0.700, 0.601966666667), (0.800, 0.501111111111), (0.900, 0.3598), (1.000, 4.4408920985e-16)

UNITS: Dimensionless

LSMLOOPfarmerProfit = IF LSMLOOPperfarmerRev=0 THEN 0 ELSE (LSMLOOPperfarmerRev-(DailyAggregationComperfarmer+DailyPerFarmerTransportCost+DailyMarketFeePerFarmerLOOP))

UNITS: taka

LSMLOOPFarmerProfitPerUnit = IF LOOPLSMOutflow=0 OR LSMLOOPfarmerProfit=0 THEN 0 ELSE (LSMLOOPfarmerProfit*LSMProportionLOOP*TrustAndCapacityAdjustedLOOPFarmers)/LOOPLSMOutflow

UNITS: Taka/Kg/Person

LSMLOOPperfarmerRev = IF (TrustAndCapacityAdjustedLOOPFarmers*LSMProportionLOOP)=0 THEN 0 ELSE LOOPRevenueLSM/(TrustAndCapacityAdjustedLOOPFarmers*LSMProportionLOOP)

UNITS: taka

LSMNLFarmerProfitPerUnit = IF LSMNLOutFlow=0 THEN 0 ELSE NLperfarmerprofitLSM*NLProportionLSM*NLFarmersupplyingday/LSMNLOutFlow

UNITS: Taka/Kg/Farmer

LSMNLOutFlow = LSM0utflow-LOOPLSMOutflow

UNITS: Kg

LSMPrefUse = IF LSMPref > 1 THEN 1 ELSE LSMPref

LSMProportionLOOP = IF LCMSupplyScenario=1 THEN 0 ELSE LSMPrefUse*ProportionToRemainingMarket

UNITS: Dimensionless

LSMPropotion = IF TotalMarketInflow=0 THEN 0 ELSE (TotalLSMflow*100)/TotalMarketInflow

UNITS: Dimensionless

LSMRevenue = LSM0utflow*TraderbuyingpriceLSM

UNITS: taka/kg

LSMTcapacity = 1400

UNITS: kg

LSMWastage = 0.05

UNITS: Percent

LTWastageRate = 0.05

UNITS: Dimensionless

MaintanenceCostPerTrip = DailyMaintainenceCost*1.5

MaintanenceCostPerTrip_A = DailyMaintainenceCost_A*1.5

UNITS: taka

MarketCost = 0.50

UNITS: taka/kg

MarketcostLCM = 0.50

UNITS: taka

MarketCostLLM = 0.50

UNITS: Taka/Kg

MarketcostLSM = 0.50

UNITS: taka/kg

MarketPriceTime = 7

UNITS: Days

MarketTrustTime = 30

UNITS: Dimensionless

Maximum_Consumption = 6.3

UNITS: Kg/day

DOCUMENT: The maximum demand for the commodity, no matter how low price goes. Units = Units / Year

MaxLocalMarketTrust = MAX(LCMTrust, LSMIVTrust)

UNITS: Dimensionless

MaxLOOPTrust = IF (((DefaultLOOPTrust+RANDOM(-RandomSelfSupplyPro, RandomSelfSupplyPro, 220))*EffectofExpectedProfitOnLOOPTrust))>1 THEN 1 ELSE ((DefaultLOOPTrust+RANDOM(-RandomSelfSupplyPro, RandomSelfSupplyPro, 220))*EffectofExpectedProfitOnLOOPTrust)

UNITS: Dimensionless

MinLOOPLLMPref = DefaultMinLOOPLLMPref/EffectofExpectedProfitOnMinLOOPLLM

UNITS: Dimensionless

MonthlyMaintainenceCost = 7000

MonthlyMaintainenceCost_A = 2000

UNITS: taka

NLAbsulateDaysPerSeasonKharif1 = IF Season = 1 THEN 92 ELSE 0

NLAbsulateDaysPerSeasonKharif2 = IF Season=2 THEN 122 ELSE 0

NLAbsulateDaysPerSeasonRabi = IF Season =3 THEN 155 ELSE 0

NLAdjustedYeild = (BaseYield*NLYieldImprovement)+BaseYield

UNITS: kg

NLConsumptionperHH = 0.07

UNITS: kg

NLDailyMarketFeePerFarmer = (NLYearlyMarketwastage*ExpectedPriceLocal)/365

UNITS: taka

NLDailyMarketWastages = NLDholta+NLTola

UNITS: Dimensionless

NLDailySupplyPerFarmer = 150

UNITS: kg

NLDailyTransportCost = (NLYearlyTransportCost/365)+TimeSavingCost

UNITS: taka

NLDholta = .10

UNITS: Dimensionless

NLfarmerProfitLCM = NLfarmerRevenuetLCM-(NLDailyMarketFeePerFarmer+NLDailyTransportCost)

UNITS: taka

NLFarmerRevenue = FarmerRevenueLLM*(1-ProportionLOOPLLMSS)

UNITS: taka

NLfarmerRevenuetLCM = IF (NLFarmersupplyingday*LCMProportionNL)=0 THEN 0 ELSE NLfarmerRevLCM/(NLFarmersupplyingday*LCMProportionNL)

UNITS: taka

NLfarmerRevLCM = LCMfarmerrevenue*(1-ProportionLOOPSSLCM)

UNITS: taka

NLFarmerRevLSM = LSMRevenue*(1-ProportionLOOPSSLSM)

UNITS: taka

NLFarmersLCM = NLFarmersupplyingday*LCMProportionNL

UNITS: Farmer

NLFarmersupplyingday = (NonLOOPFarmers/FarmerSupplyFreNL)+LOOPNLSupplyingPerDay

UNITS: People

NLFertilizer = LOGNORMAL(12872,8418, 2)/YearToDayConverter

UNITS: taka/day

NLGivenawayPerHH = 0.05

UNITS: Dimensionless

NLHarvesting = LOGNORMAL(19.4, 17.3, 23)*LabourHourlyWage

UNITS: taka

NLHilling = LOGNORMAL(3.95, 5.15, 19)*LabourHourlyWage

UNITS: taka

NLHormone = LOGNORMAL(1137, 969, 6)/YearToDayConverter

UNITS: taka/day

NLIrrigation = LOGNORMAL(3171, 2066, 4 )/YearToDayConverter

UNITS: taka/day

NLLandHoldingDuringKharif = IF Season=2 THEN 0.001 ELSE 0

UNITS: Decimal

NLLCMOutFlow = LCMoutflow-LOOPLCMOutflow

UNITS: kg

NLLCMWastage = 0.10

UNITS: Percent

NLManure = LOGNORMAL(2049, 1623, 5)/YearToDayConverter

UNITS: taka/day

NLNetWieghtedProfitPerUnit = (WeightedAvprofitperNLfarmerPerUnit-PerUnitInputcostNL)

UNITS: Taka/Kg

NLOthers = LOGNORMAL(3.08, 4.19, 24)*LabourHourlyWage

UNITS: taka

NLPerDayCost = SMTH1(NLSeeds+NLFertilizer+NLPesticides+NLIrrigation+NLManure+NLTractor+NLRota+NLHormone, 30)

UNITS: taka

NLperdaylaborcost = (NLPloughing+NLPlanting+NLHilling+NLWeeding+NLWatering+NLHarvesting+NLOthers+NLpest)/YearToDayConverter

UNITS: taka

NLperfarmerprofitLSM = NLPerfarmerRevLSM-(NLDailyMarketFeePerFarmer+NLDailyTransportCost)

UNITS: taka

NLPerFarmerRev = IF NLFarmerRevenue=0 THEN 0 ELSE (NLFarmerRevenue/(NLFarmersupplyingday*NLProportionLLM))

UNITS: taka

NLPerfarmerRevLSM = IF (NLFarmersupplyingday*NLProportionLSM)=0 THEN 0 ELSE NLFarmerRevLSM/(NLFarmersupplyingday*NLProportionLSM)

UNITS: taka

NLPerFarmerVolume = NLProductionOutflow/NLFarmersupplyingday

UNITS: kg

NLpest = LOGNORMAL(.90, 2.64, 22)*LabourHourlyWage

UNITS: taka

NLPesticides = LOGNORMAL(6375, 5342, 3)/YearToDayConverter

UNITS: taka/day

NLPlanting = LOGNORMAL(3.8, 4.15, 18)*LabourHourlyWage

UNITS: taka

NLPloughing = LOGNORMAL(3.68, 3.78, 17)*LabourHourlyWage

UNITS: taka

NLProportionLLM = 0.80

UNITS: Dimensionless

NLProportionLSM = 0.10

UNITS: Percent

NLRota = LOGNORMAL(429, 524, 8)/YearToDayConverter

UNITS: taka/day

NLSalesPerFarmer = TotalNLSales/NLFarmersupplyingday

UNITS: Kg

NLSeasonalProfitChange = CumulativeperNL_farmerProfit-DELAY(CumulativeperNL_farmerProfit, NumberOfDaysPerSeasonNL)

NLSeeds = LOGNORMAL(3844, 3704, 1)/YearToDayConverter

UNITS: taka/day

NLTola = .04

UNITS: Dimensionless

NLtotaldaycost = NLPerDayCost+NLperdaylaborcost

UNITS: taka

NLTotalRev = NLFarmerRevLSM + NLFarmerRevenue + NLfarmerRevLCM

UNITS: kg

NLTractor = LOGNORMAL(1304, 980, 7)/YearToDayConverter

UNITS: taka/day

NLTransportCost = 1

UNITS: taka/kg

NLtravellingcost = NLtravellingtime/60*100

UNITS: taka

NLtravellingtime = 90

UNITS: Minutes

NLWatering = LOGNORMAL(1.14, 4.31, 21)*LabourHourlyWage

UNITS: taka

NLWeeding = LOGNORMAL(15.23, 15.57, 20)*LabourHourlyWage

UNITS: taka

NLWeightedProfitPerFarmer = (((NLperfarmerprofitLSM*NLProportionLSM)+(LLMPerFarmerProfitNL*NLProportionLLM)+(NLfarmerProfitLCM*LCMProportionNL)))-TotalNLdaycost

UNITS: taka

NLWieghtedDailyProfitPerFarmer = NLWeightedProfitPerFarmer

UNITS: taka

NLYearlyMarketwastage = NLYearlySupplyPerfarmer*NLDailyMarketWastages

UNITS: kg

NLYearlySupplyPerfarmer = YearlyMarketSupplyFre*NLDailySupplyPerFarmer

UNITS: kg

NLYearlyTransportCost = CostPerRV*YearlyMarketSupplyFre

UNITS: taka

NLYeildInvestment = YieldInvestment/NumberOfDaysPerSeasonNL

UNITS: taka

NLYieldImprovement = GRAPH(InvestmentproportionCostNL)

NLYieldinvestmentRate = 0.02

UNITS: taka

NonLOOPAggregationCost = NLTransportCost+PerKGNonLOOPTravelingCost

UNITS: taka

NonLOOPFarmers = (Population-LOOPFarmers)

UNITS: People

nooffarmersperaggregator = 20

UNITS: Farmer

NumberOfAggregators = LOOPFarmersSupplyingDay/nooffarmersperaggregator

UNITS: People

NumberOfDaysPerSeason = AbsulateDaysPerSeasonKharif1+AbsulateDaysPerSeasonKharif_2+AbsulateDaysPerSeasonRabi

NumberOfDaysPerSeasonNL = NLAbsulateDaysPerSeasonKharif1+NLAbsulateDaysPerSeasonKharif2+NLAbsulateDaysPerSeasonRabi

PerAggreDailyTransaction = IF NumberOfAggregators=0 THEN 0 ELSE LOOPAggregationOutflow/NumberOfAggregators

UNITS: kg

PerAggreDailyTransportCost = IF TIME <=309 THEN (PerKGTAlamshadhuCostLCM*PerAggreDailyTransaction-(PerKGTAlamshadhuCostLCM*PerAggreDailyTransaction*0.5)) ELSE PerKGTAlamshadhuCostLCM*PerAggreDailyTransaction

UNITS: taka

PerceivedInventoryCoverage = SMTH1(InventoryCoverageLLM, CoveragePerceptionTime)

UNITS: Dimensionless

PerceiveinventorycoverageLCM = SMTH1(InventoryCoverageLCM, CoverageperceptiontimeLLCM)

UNITS: Dimensionless

PerceiveinventorycoverageLSM = SMTH1(InventoryCoverageLSM, CoverageperceptiontimeLLSM)

UNITS: Dimensionless

PerDayLOOPVolumefrequency = LOOPPerFarmerVolume

UNITS: kg

PerFarmerLSMTransportCost = PerFarmerSupplyingLSM*TranportCostPerUnitPerFarmer

UNITS: taka

PerFarmerSupplyingLSM = LSMSupplyLOOP/FarmerSuppllyingLSM

UNITS: kg

PerHourCost = 80

UNITS: taka

PerKGDholtaLLMCost = TradersBuyingPriceLLM*DholtaLLM

UNITS: taka

PerKgDholtaLSM = TraderbuyingpriceLSM*DholtaLSM

UNITS: taka

PerKgLaborcostLCM = 1

UNITS: taka/kg

PerKgLaborCostLLM = 1

UNITS: Taka/Kg

PerKgLaborcostLSM = 1

UNITS: taka/kg

PerKGLabourCost = BuyingCapacity/TotalLabourCost

PerKGNonLOOPTravelingCost = NLtravellingcost/Volumeperfarmer

PerKGTAlamshadhuCostLCM = CostPerTripAlamshadhu/AlamshadhuCapacity

PerKGtolaLLMCost = TradersBuyingPriceLLM*TolaLLM

UNITS: taka

PerKGtolaLSMcost = TraderbuyingpriceLSM*TolaLSM

UNITS: taka

PerKGtotalLCMCost = TraderbuyingpriceLCM*TolaLCM

UNITS: taka

PerKGTransportCost = TransportcostPerTripTruck/VehicleCapacity

PerRetailerVegExpenditure = RetailerVegExpenditure/RetailerLCM

UNITS: taka

PerTripConsumption = Distancecovered/DiegelCost

UNITS: km/litre

PerTripDepriciationCost = DailyDepreciation*2

PerTripDepriciationCost_A = DailyDepreciation_A*2

UNITS: taka

PerTripFuelConsumption = UpstreamMarketDistance/FuelEconomy

UNITS: km/litre

PerTripProfit = 500

UNITS: taka

PerUnitCostLOOPReduction = PerUnitInputCostLOOP-(PerUnitInputCostLOOP*LOOPCostReductionRate)

UNITS: Taka/Kg

PerUnitInputCostLOOP = (LOOPtotaldaycost/PerDayLOOPVolumefrequency)

UNITS: Taka/Kg

PerUnitInputcostNL = NLtotaldaycost/PerDayLOOPVolumefrequency

UNITS: Taka/Kg

PriceTrustTime = 7

UNITS: Days

ProductionPerLoopFarmer = LOOPProductionAvaiableForAggregation/LOOPFarmers

ProductionPerNLFarmer = NLproductioninflow/NonLOOPFarmers

Profitoftraders = (ExpectedSellingprice-TradersBuyingcost)

UNITS: taka/kg

ProfitPerceptionTime = 120

UNITS: Days

ProfitPerDT = (ActualSalesPriceLLM-TradersBuyingPriceLLM)

UNITS: taka

ProfitPerretailer = RevenuePerRetailer-(PerRetailerVegExpenditure+TotalPerRetailerSalesCost)

UNITS: taka

ProfitPerTrip = 3000

ProportionLOOPLLMSS = IF (LOOPLLMInFlow+NLLLMinflow)=0 THEN 0 ELSE LOOPLLMInFlow/(LOOPLLMInFlow+NLLLMinflow)

UNITS: Dimensionless

ProportionLOOPSSLCM = IF (LCMLOOPInFlow+NLLCMflow)=0 THEN 0 ELSE LCMLOOPInFlow/(LCMLOOPInFlow+NLLCMflow)

UNITS: Dimensionless

ProportionLOOPSSLSM = IF (LSMSupplyLOOP+NLLSMinflow)=0 THEN 0 ELSE LSMSupplyLOOP/(LSMSupplyLOOP+NLLSMinflow)

UNITS: Dimensionless

ProportionToRemainingMarket = (1-LLMProportionLOOP)

UNITS: Dimensionless

RabiSupplyFreq = IF Season = 3 THEN 3 ELSE 0

UNITS: days

RabiYeildPerSeason = IF Season=3 THEN 105 ELSE 0

UNITS: Kg/Decimal

RandomLOOPprice = RANDOM(15, 20, 12)

UNITS: taka/kg

RandomNLprice = RANDOM(15, 20, 13)

UNITS: taka/kg

RandomSelfSupplyPro = 0.05

UNITS: Dimensionless

Ratiofertilizercost = LOOPtotaldaycost/LOOPFertilizer

UNITS: %

RationOfProfitCost = LOOPWieghtedDailyProfitPerFarmer/LOOPtotaldaycost

UNITS: Dimensionless

Reference_Consumer_Demand = 2.60

UNITS: Kg/day

DOCUMENT: Initial value of customer orders. Units = Units/Year

Reference_consumer_Demand_Elasticity = 0.58

UNITS: Dimensionless

DOCUMENT: Demand elasticity at the reference price

Dimensionless

Semi -urban

file:///C:/Users/gc25/Downloads/--1465997973-38.IJASR-DemandSupplyTradeProspectsofMajorFruits.pdf

Reference_Price = 25

UNITS: taka

DOCUMENT: Price at which industry demand equals reference demand.

Units = $/Unit

RelativeCost = IF LOOPDailyAggrega_tionCostPerFarmer>NLDailyTransportCost THEN 0.2 ELSE -0.2

UNITS: Dimensionless

RelativewastageCost = NLDailyMarketFeePerFarmer/DailyMarketFeePerFarmerLOOP

UNITS: Dimensionless

RetailerLCM = GRAPH(TIME)

RetailerMarginGraphical = GRAPH(PerceiveinventorycoverageLCM)

(0.000, 7.395), (2.000, 0.000)

UNITS: Taka/Kg

RetailerRevenue = RetailerSellingPrice*LCMoutflow

UNITS: taka

RetailerSellingPrice = IF TIME=1 THEN 25 ELSE RetailerMarginGraphical+TraderbuyingpriceLCM

UNITS: Taka/Kg

RetailerTranspostCost = 0.5

UNITS: Taka/kg

RetailerVegExpenditure = LCMoutflow*TraderbuyingpriceLCM

UNITS: taka

RevenuePerKGLCM = TraderbuyingpriceLCM-(PerKGtotalLCMCost+TransportcostPerKgLCM)

UNITS: taka

RevenuePerKGLLM = TradersBuyingPriceLLM-(TransportcostPerKGLLM+PerKGtolaLLMCost+PerKGDholtaLLMCost)

UNITS: taka

RevenuePerKGLSM = TraderbuyingpriceLSM-(PerKGtolaLSMcost+PerKgDholtaLSM+TransportcostPerKgLSM)

UNITS: taka

RevenuePerRetailer = RetailerRevenue/RetailerLCM

UNITS: taka

Season = GRAPH(TIME)

seasonalLLM = GRAPH(TIME)

(1.0, 345.1), (31.3333333333, 288.1), (61.6666666667, 255.6), (92.0, 216.5), (122.333333333, 118.8), (152.666666667, 89.5), (183.0, 104.2), (213.333333333, 185.6), (243.666666667, 175.8), (274.0, 153.0), (304.333333333, 195.3), (334.666666667, 320.7), (365.0, 320.7)

UNITS: People

SeasonChange = IF (NumberOfDaysPerSeason- DELAY(NumberOfDaysPerSeason, 1))=0 THEN 0 ELSE 1

SeasonChangeNL = IF (NumberOfDaysPerSeasonNL- DELAY(NumberOfDaysPerSeasonNL, 1))=0 THEN 0 ELSE 1

SensivityOfPriceCostLCM = 0.7

UNITS: Dimensionless

SensivityOfPriceCostLSM = 0.7

UNITS: Dimensionless

SensivityOfPriceCots = 0.7

UNITS: Dimensionless

SensivityOfPriceto_InventoryCoverage = -0.2

UNITS: Dimensionless

SensivityOfPriceto_InventoryCoverageLCM = -0.6

UNITS: Dimensionless

SensivityOfPriceto_InventoryCoverageLSM = -0.6

UNITS: Dimensionless

SmallMarketLongRunExpectedProfitability = (LongRunRevPerKgSmallMarket-LongRunRevPerKgLLM)/LongRunRevPerKgSmallMarket

UNITS: Dimensionless

SupplyPerFarmerPerDay = (LOOPAggregationOutflow/TrustAndCapacityAdjustedLOOPFarmers)

UNITS: kg

SupplyPreferenceLOOP = IF(LOOPAggregationCost>NonLOOPAggregationCost) THEN 0.2 ELSE 0.8

SupplySwitch = 0

UNITS: Dimensionless

ThreshholdProfitforColdstorage = 20

UNITS: Taka/Kg

TimeSave = RANDOM(4, 5, 101)

UNITS: Hours

TimeSavingCost = TimeSave*PerHourCost

UNITS: taka

TolaLCM = 0.04

UNITS: Percent

TolaLLM = 0.04

UNITS: Percent

TolaLSM = 0.04

UNITS: Percent

TollFee = RANDOM(1000, 1500, 102)/5

UNITS: taka

TotalCost = DailyAggregationComperfarmer + DailyMarketFeePerFarmerLOOP + LOOPDailyTransportcostPerfarmers + LOOPtotaldaycost

UNITS: taka

TotalDailyDD = LLMDemand + LCMDailyDemand + LSMdemand

UNITS: kg/day

TotalDays = 365

UNITS: Days

TotalLabourCost = LabourRequired*LabourRate

UNITS: taka

TotalLCMflow = NLLCMflow+LCMLOOPInFlow

UNITS: kg

TotalLCMOutFlow = NLLCMOutFlow+LOOPLCMOutflow

UNITS: kg

TotalLLMinflow = LOOPLLMInFlow+NLLLMinflow

TotalLOOPcost = LOOPtotaldaycost++dailyloancost

UNITS: taka

TotalLOOPMarketInflow = LCMLOOPInFlow + LOOPLLMInFlow + LSMSupplyLOOP

UNITS: kg

TotalLOOPPdn = LOOPFarmers*LOOPAdjustedYeild*LandStockPerFarmer*VegLandProportionlimit

UNITS: kg

TotalLOOPSales = LOOPLCMOutflow + LOOPLLMOutflow + LOOPLSMOutflow

UNITS: kg

TotalLSMflow = NLLSMinflow+LSMSupplyLOOP

UNITS: kg

TotalLSMOutFlow = LOOPLSMOutflow+LSMNLOutFlow

UNITS: kg

TotalLSMtraders = "#ofMarket"*"#OfTradersLSM"

UNITS: People

TotalMarketInflow = LCMLOOPInFlow + LOOPLLMInFlow + LSMSupplyLOOP + NLLCMflow + NLLLMinflow + NLLSMinflow

UNITS: kg

TotalMarketsupplyLOOP = LCMLOOPInFlow + LOOPLLMInFlow + LSMSupplyLOOP

UNITS: kg

TotalNLdaycost = NLPerDayCost+dailyloancost+NLperdaylaborcost

UNITS: taka

TotalNLSales = LLMNLOutFlow + LSMNLOutFlow + NLLCMOutFlow

UNITS: kg

TotalNLSales_1 = LLMNLOutFlow + LSMNLOutFlow + NLLCMOutFlow

UNITS: kg

TotalPerFarmerRevNL = NLTotalRev/NLFarmersupplyingday

UNITS: kg

TotalPerRetailerSalesCost = (LCMoutflow*LCMRetailerSalesCost)/RetailerLCM

UNITS: taka

TotalSales = TotalLOOPSales+TotalNLSales

UNITS: kg

TotalVegForMarket = HHEatenOutFlow + HHGivenAwayOutflow + LOOPAggregationOutflow + NonLoopoutflow

UNITS: kg

TPerFarmerPerUnitRev = IF TotalPerFarmerRevNL=0 THEN 0 ELSE TotalPerFarmerRevNL/TotalNLSales_1

UNITS: Taka/Kg

TraderbuyingpriceLCM = EffectiveOfInvent_oryCoverageOnPriceLCM*EffecttocostsonpriceLCM*LCMexpectedprice

UNITS: Dimensionless

TraderbuyingpriceLSM = EffectiveOfInvent_oryCoverageOnPriceLSM*EffecttocostsonpriceLSM*LSMexpectedprice

UNITS: Dimensionless

TradersBuyingcost = Costofproduce+WastageCost+MarketCost+PerKGLabourCost+PerKGTransportCost

UNITS: taka

TradersBuyingPriceLLM = IF TIME <=540 THEN TradersExpectedPrice*EffectofCotsOnPrice*EffectiveOfInvent_oryCoverageOnPrice ELSE (TradersExpectedPrice*EffectofCotsOnPrice*EffectiveOfInvent_oryCoverageOnPrice)/1

UNITS: taka/kg

TradersExpectedPrice = ActualSalesPriceLLM

UNITS: taka/kg

TranportCostPerUnitPerFarmer = IF TIME >=540 THEN ((CostPerDayPerAggregator/(PerAggreDailyTransaction))*(1-TransportSubsidyAll%)) ELSE (CostPerDayPerAggregator/(PerAggreDailyTransaction))

UNITS: Taka/Kg

TransportcostPerKgLCM = 0.68

UNITS: taka

TransportcostPerKGLLM = 0.63

UNITS: taka

TransportcostPerKgLSM = 0.86

UNITS: taka

TransportcostPerTripTruck = FerryCost+TollFee+FuelCostPerTrip+MaintanenceCostPerTrip+PerTripDepriciationCost+DriverCost+ProfitPerTrip

UNITS: taka

TransportSubsidyAll% = IF TransportSubsidySwitchAll=0 THEN 0 ELSE 0.5

UNITS: Dimensionless

TransportSubsidyLCM% = IF TransportSubsidySwitchLCM =0 THEN 0 ELSE 0.5

UNITS: Dimensionless

TransportSubsidySwitchAll = 0

TransportSubsidySwitchLCM = 0

UNITS: Dimensionless

Tripdays = RANDOM(1, 3, 257)

UNITS: Days

TrustAndCapacityAdjustedLOOPFarmers = LOOPFarmersSupplyingDay*MaxLOOPTrust*(1-LoopCapacityLimitedFarmersProp)

UNITS: Dimensionless

TwoseasonModel = GRAPH(TIME)

VegeatenPerHH = HHEatenOutFlow/LOOPFarmers

UNITS: kg

VegetablesCostLCM = RANDOM(15, 20, 303)

UNITS: taka/kg

VegetablesCostLSM = RANDOM(15, 20, 302)

UNITS: taka/kg

VegetablesPerKgCostLLM = RANDOM(15, 20, 301)

VegLandProportionlimit = IF VegLandProportion>1 THEN 1 ELSE VegLandProportion

UNITS: Dimensionless

VegLandProportionlimitNL = IF VegLandProportionNL>1 THEN 1 ELSE VegLandProportionNL

UNITS: Dimensionless

Vehicle#PerDay = IF AlamshadhuCapacity/(1+PerAggreDailyTransaction)<=1 THEN 1 ELSE 2

UNITS: kg

VehicleCapacity = 6000

UNITS: kg

VehicleLifetime = 15

VehiclePrice = 1500000

Volumeperfarmer = 250

UNITS: kg

WastageCost = 1

UNITS: taka/kg

WastagecostLCM = 1

UNITS: taka/kg

WastageCostLLM = 1

UNITS: Taka/Kg

WastagecostLSM = 1

UNITS: taka/kg

WastageLLM = 0.10

UNITS: Percent

WeightedAvprofitperLOOPfarmerPerUnit = IF TotalLOOPSales=0 THEN 0 ELSE (LOOPLLMPerFarmerProfitPerUnit*(LOOPLLMOutflow/TotalLOOPSales)+LSMLOOPFarmerProfitPerUnit*(LOOPLSMOutflow/TotalLOOPSales)+LCMLOOPFarmerProfitPerUnit*(LOOPLCMOutflow/TotalLOOPSales))

UNITS: taka/Kg/Farmer

WeightedAvprofitperNLfarmerPerUnit = IF TIME <=7 THEN 15 ELSE ((LLMNLProfitPerUnit*NLProportionLLM)+(LCMNLPerfarmerProfitPerUnit*LCMProportionNL)+(LSMNLFarmerProfitPerUnit*NLProportionLSM))

UNITS: taka/kg/farmer

WeightedTotalProfitPerFarmer = ((LOOPWieghtedDailyProfitPerFarmer*TotalLOOPSales)/(TotalLOOPSales+TotalNLSales))+((NLWieghtedDailyProfitPerFarmer*TotalNLSales)/(TotalNLSales+TotalLOOPSales))

UNITS: Taka/Day

WordOfMouthEffectiveness = 0.0001

UNITS: Dimensionless

YearlyDepriciationCost = VehiclePrice/VehicleLifetime

YearlyDepriciationCost_A = AlamshadhuPrice/Alamshadhulifetime

YearlyMarketSupplyFre = 96

UNITS: Dimensionless

YearlyMarketwastage = YearlySupplyPerfarmer*DailyMarketWastages

UNITS: kg

YearlySupplyPerfarmer = YearlyMarketSupplyFre*LOOPPerFarmerVolume

UNITS: kg

YearToDayConverter = 365

UNITS: day

Yieldinvestment_rate = 0.1

UNITS: taka

{The model has 586 (586) variables (array expansion in parens).

In root model and 0 additional modules with 8 sectors.

Stocks: 33 (33) Flows: 56 (56) Converters: 497 (497)

Constants: 129 (129) Equations: 424 (424) Graphical: 22 (22)

There are also 65 expanded macro variables.}
